# Early warning scores for detecting deterioration in adult hospital patients: systematic review and critical appraisal of methodology

**DOI:** 10.1136/bmj.m1501

**Published:** 2020-05-20

**Authors:** Stephen Gerry, Timothy Bonnici, Jacqueline Birks, Shona Kirtley, Pradeep S Virdee, Peter J Watkinson, Gary S Collins

**Affiliations:** 1 Centre for Statistics in Medicine, Nuffield Department of Orthopaedics, Rheumatology and Musculoskeletal Sciences, University of Oxford, Oxford OX3 7LD, UK; 2 Critical Care Division, University College London Hospitals NHS Trust, London, UK; 3 Oxford University Hospitals NHS Foundation Trust, Oxford, UK; 4 Nuffield Department of Clinical Neurosciences, University of Oxford, Oxford, UK

## Abstract

**Objective:**

To provide an overview and critical appraisal of early warning scores for adult hospital patients.

**Design:**

Systematic review.

**Data sources:**

Medline, CINAHL, PsycInfo, and Embase until June 2019.

**Eligibility criteria for study selection:**

Studies describing the development or external validation of an early warning score for adult hospital inpatients.

**Results:**

13 171 references were screened and 95 articles were included in the review. 11 studies were development only, 23 were development and external validation, and 61 were external validation only. Most early warning scores were developed for use in the United States (n=13/34, 38%) and the United Kingdom (n=10/34, 29%). Death was the most frequent prediction outcome for development studies (n=10/23, 44%) and validation studies (n=66/84, 79%), with different time horizons (the most frequent was 24 hours). The most common predictors were respiratory rate (n=30/34, 88%), heart rate (n=28/34, 83%), oxygen saturation, temperature, and systolic blood pressure (all n=24/34, 71%). Age (n=13/34, 38%) and sex (n=3/34, 9%) were less frequently included. Key details of the analysis populations were often not reported in development studies (n=12/29, 41%) or validation studies (n=33/84, 39%). Small sample sizes and insufficient numbers of event patients were common in model development and external validation studies. Missing data were often discarded, with just one study using multiple imputation. Only nine of the early warning scores that were developed were presented in sufficient detail to allow individualised risk prediction. Internal validation was carried out in 19 studies, but recommended approaches such as bootstrapping or cross validation were rarely used (n=4/19, 22%). Model performance was frequently assessed using discrimination (development n=18/22, 82%; validation n=69/84, 82%), while calibration was seldom assessed (validation n=13/84, 15%). All included studies were rated at high risk of bias.

**Conclusions:**

Early warning scores are widely used prediction models that are often mandated in daily clinical practice to identify early clinical deterioration in hospital patients. However, many early warning scores in clinical use were found to have methodological weaknesses. Early warning scores might not perform as well as expected and therefore they could have a detrimental effect on patient care. Future work should focus on following recommended approaches for developing and evaluating early warning scores, and investigating the impact and safety of using these scores in clinical practice.

**Systematic review registration:**

PROSPERO CRD42017053324.

## Introduction

Research towards the end of the 20th century showed the incidence of adverse events and unnecessary deaths in hospital patients.^1^ ^2^ ^3^ ^4^ Early warning scores (EWSs) were proposed as a potential solution.^5^ These tools are clinical prediction models that generally use measured vital signs to monitor patients’ health during their hospital stay. The models identify the likelihood of patients deteriorating, which is often defined as death or admission to the intensive care unit. When a patient shows signs of deterioration, the EWS triggers a warning so that care can be escalated. Historically EWSs were implemented on paper based observation charts, but now they are increasingly becoming part of electronic health record systems.

EWSs based on vital signs are widely used every day in hospitals to identify patients who are clinically deteriorating. These measures are routinely used in several countries, including the Netherlands, the United States, Australia, and the Republic of Ireland.^6^ ^7^ ^8^ ^9^ In hospitals in the United Kingdom EWS use is mandated as a standard of care by the National Institute for Health and Care Excellence.^10^ Because hospital inpatients are usually assessed every few hours by using an EWS, these scores are used hundreds of millions of times each year.^11^ Requests have also been made to increase EWS use across ambulance services, primary care, and community care homes.^12^ ^13^ ^14^ ^15^ ^16^

Articles that describe the development of clinical prediction models abound in many areas of medicine.^17^ ^18^ Systematic reviews have shown that the methods used in these papers are often poor.^19^ ^20^ ^21^ ^22^ Although many published prediction models are not put into practice, EWSs are used widely, probably more than any other type of clinical prediction model. Despite extensive development and increasing uptake, comprehensive reviews of EWS articles in the past decade have been lacking. Systematic reviews are needed that assess the methodological and reporting quality of papers describing the development and validation of EWSs. External validation studies, which are vital for assessing the generalisability of EWSs, need to be systematically evaluated. Existing systematic reviews of EWSs have mostly concentrated on predictive performance, but have hinted at methodological flaws.^23^ ^24^

Hospital patients will probably have their vital signs and other parameters measured several times during their hospital stay, therefore the available datasets might include multiple measurements (or observation sets) for each patient. The most appropriate way to analyse such data is not clear, which increases the complexity of EWS research in comparison to other areas of clinical prediction modelling.^25^ ^26^ Debate also exists about the best choice of outcome measure and time horizon; for example, death or admission to intensive care within a specific time period (eg, 24 hours) or the whole hospital stay.^27^ Different approaches to these problems might give different results when developing and validating EWSs, and could lead to models being used that do not work.

The great potential for EWSs to assist in clinical decision making might be thwarted by poor methods and inadequate reporting. The widespread use of EWSs means poorly developed and reported EWSs could have a highly detrimental effect on patient care. We carried out a systematic review to assess the methods and reporting of studies that developed or externally validated EWSs for general adult patients.

## Methods

Details of the study design and rationale have been previously published.^11^ In summary, we identified articles that described the development or validation of EWSs. The Medline (Ovid), CINAHL (EBSCOHost), PsycInfo (Ovid), and Embase (Ovid) databases were searched from inception to 30 August 2017. An update search was conducted on 19 June 2019 to identify articles published since the date of the original search. Search strategies were developed by an information specialist (SK) for each database and are reported in the supplementary appendix. Search terms included relevant controlled vocabulary terms (eg, MeSH, EMTREE) and free text variations for early warning or track and trigger scores or systems (including common acronyms), physiological monitoring or health status indicators, combined with development and validation terms. We did not apply any date or language restrictions to the search. Additional articles were found by searching the references in papers identified by the search strategy, our own personal reference lists, and a Google Scholar search.

### Eligibility criteria

We included any primary research articles that described the development or validation of one or more EWSs, defined as a score (with at least two predictors) used to identify general patients admitted to hospital who are at risk of clinical deterioration. External validation studies were only included if an article describing the development of that EWS was also available.

Articles were not eligible if the score was developed for use in a subset of patients with a specific disease or group of diseases, for use in children (<16 years old), or in pregnant women; when the score is intended to be used for outpatients or for patients in the intensive care unit; when no vital signs were included in the final model; or when the article was a review, letter, personal correspondence or abstract, or the article was published in a non-English language.

### Study selection and data extraction

One author (SG) screened the titles and abstracts of all articles identified by the search string. Two reviewers (from SG, PSV, and JB) independently extracted data by using a standardised and piloted data extraction form. Conflicts were resolved by discussion between the two relevant reviewers. The form was administered by using the REDCap (research electronic data capture) electronic data capture tool.^28^ The items for extraction were based on the CHARMS (critical appraisal and data extraction for systematic reviews of prediction modelling studies) checklist,^29^ supplemented by subject specific questions and methodological guidance. These items included study design characteristics, patient characteristics, sample size, outcomes, statistical analysis methods, and model performance methods.

Items extracted from studies describing the development of EWSs included the following (for an explanation of some of the technical terms, see box 1): study design (retrospective, prospective), details of population (eg, when and where data were collected, age, sex), method of development (eg, clinical consensus, statistical approach), predicted outcome and time horizon, number and type of predictors, sample size, number of events, missing data approach, modelling approach (eg, type of regression model, method used to select variables, handling of continuous variables, examination of interaction terms), model presentation (eg, reporting of model coefficients, intercept or baseline hazard, simplified model), method of internal validation (eg, split sample, bootstrapping, cross validation), and assessment of model performance (eg, discrimination, calibration). Items extracted from studies describing the external validation of EWSs included study design (retrospective, prospective), details of population (eg, when and where data were collected, age, sex), predicted outcome and time horizon, sample size, number of events, missing data approach, and assessment of model performance (eg, discrimination, calibration). We define event patients as the number of patients recorded as having the outcome of interest (eg, dying or being admitted to the intensive care unit at any point during their hospital stay). Event observations refer to the number of observation sets that are within a defined period before the outcome occurs.

Box 1 **Definitions of technical terms**

Apparent performance: evaluation of the model’s predictive accuracy with the same data used to develop it
Internal validation: evaluation of the model’s predictive accuracy in the population in which the model is intended for use; the apparent performance is adjusted for the optimism resulting from overfitting
External validation: evaluation of the model’s predictive accuracy with data other than those used to develop it
Discrimination: ability of the model to distinguish between patients who will and those who will not go on to develop the outcome of interest; typically measured using the C index
Calibration: agreement between predicted risks and observed event rates
Prediction horizon: timeframe in which the model is intended to predict the outcome of interest
Individualised risk prediction: ability of the model to estimate probability of outcome occurring based on patient’s characteristics
Observation set: vital sign measurements of an individual patient at a particular point in time; typically consists of blood pressure, heart rate, respiratory rate, temperature, and oxygen saturation

### Assessment of bias

We assessed the risk of bias for each article by using PROBAST (prediction model risk of bias assessment tool), which was developed by the Cochrane Prognosis Methods Group.^30^ PROBAST consists of 23 signalling questions within four domains (participant selection, predictors, outcome, and analysis). The articles were classified as low, high, or unclear risk of bias for each domain. A study was classified as having an overall low risk of bias only if it was at low risk of bias within each domain.

### Evidence synthesis

We summarised the results by using descriptive statistics, graphical plots, and a narrative synthesis. We did not perform a quantitative synthesis of the models because this was not the main focus of the review, and the studies were too heterogeneous to combine.

### Patient and public involvement

Patients and members of the public were involved in setting the research question and developing the study, through face-to-face meetings and revisions of the protocol. Patients and members of the public have read and revised the manuscript. There are no plans to disseminate the results of the research to patients or the public.

## Results

**Fig 1:**
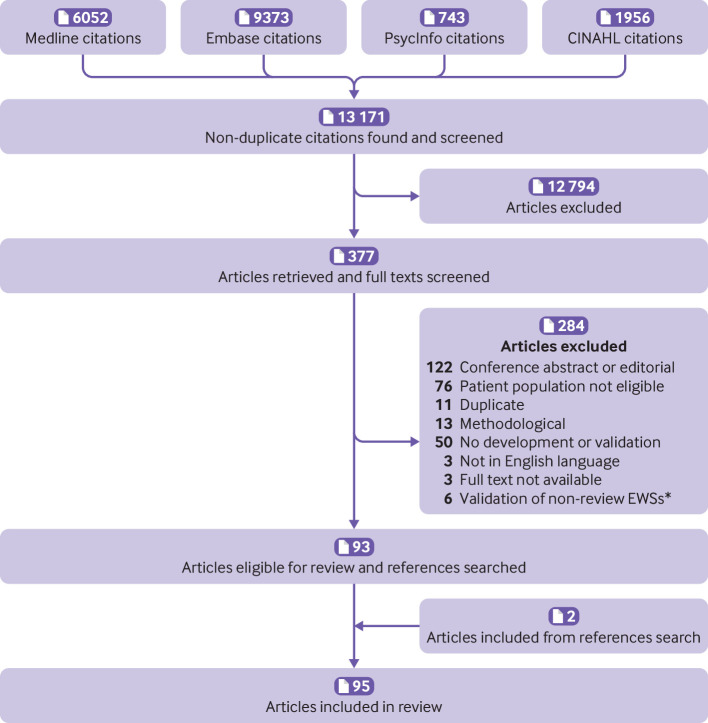
Flow diagram of article selection. *Validation of non-review EWSs (early warning scores) refers to external studies, which are excluded because the corresponding development paper was ineligible or because no development paper has been published

The search strategy identified 13 171 unique articles, of which 12 794 were excluded based on title and abstract screening. We screened 377 full texts, 93 of which met the eligibility criteria and were included in the review (fig 1). We identified two more articles by searching the article references, which were also included, giving 95 articles in total. Eleven articles described development of EWSs only, 23 described development and external validation, and 61 articles described external validation only. The articles were published between 2001 and 2019 in 51 journals. One journal, *Resuscitation*, published 21 of the articles. No other journal included more than four of the articles. Ninety three articles used a patient dataset (two used only clinical consensus), with most using data from the UK (n=28) or the US (n=25). The articles represented data from 22 countries across four continents.

### Studies describing development of EWSs

#### Study design

Of the 34 articles describing the development of a new EWS, 29 were based on statistical methods; that is, they used some form of data driven approach to create the model. Three studies developed models based on clinical consensus, where a group of experts chose the variables and associated weights that would form the model. Two studies developed models by modifying an existing score, either through modifying the variable weights or through adding binary variables, to improve predictive performance (table 1), however the rationale for modifying an existing score was not reported.

**Table 1:** Study design characteristics of 34 articles describing development of early warning score

Reference	EWS	Type of development	Type of data	Country	Years of data	Mean or median age	Male (%)
Albert 2011^31^	—	Based on clinical consensus	NA	US	NA	NA	NA
Alvarez 2013^32^	—	Using statistical methods (based on data)	Retrospective cohort/database	US	2009-10	51	56
Badriyah 2014^33^	DTEWS	Using statistical methods (based on data)	Retrospective cohort/database	UK	2006-08	68	47
Bleyer 2011^34^	Trio of critical vital signs	Using statistical methods (based on data)	Retrospective cohort/database	US	2009	57	51
Churpek 2012^8^	CART	Using statistical methods (based on data)	Retrospective cohort/database	US	2008-11	54	43
Churpek 2014^35^	—	Using statistical methods (based on data)	Retrospective cohort/database	US	2008-11	54	43
Churpek 2014^36^	eCART	Using statistical methods (based on data)	Retrospective cohort/database	US	2008-13	60	40
Churpek 2016^37^	—	Using statistical methods (based on data)	Retrospective cohort/database	US	2008-13	60	40
Cuthbertson 2010^38^	—	Using statistical methods (based on data)	Prospective cohort	UK	2005	65	51
Douw 2016^9^	DENWIS	Modification of existing score	NA	Netherlands	NA	NA	NA
Duckitt 2007^39^	Worthing PSS	Using statistical methods (based on data)	Prospective cohort	UK	2003-05	73	52
Dziadzko 2018^40^	APPROVE	Using statistical methods (based on data)	Retrospective cohort/database	US	2013	58	41
Escobar 2012^41^	EMR based model	Using statistical methods (based on data)	Retrospective cohort/database	US	2006-09	65	45
Faisal 2018^42^	CARM	Using statistical methods (based on data)	Prospective cohort	UK	2014-15	67	50
Ghosh 2018^43^	EDI	Using statistical methods (based on data)	Retrospective cohort/database	US	2012-13	59	Missing
Goldhill 2004^44^	—	Using statistical methods (based on data)	Prospective cohort	UK	2002	61	Missing
Harrison 2006^45^	GMEWS	Modification of existing score	NA	Australia	NA	NA	NA
Jones 2012^46^	NEWS	Based on clinical consensus	NA	UK	NA	NA	NA
Kellett 2006^47^	SCS	Using statistical methods (based on data)	Retrospective cohort/database	Ireland	2000-04	62	52
Kellett 2008^48^	HOTEL	Using statistical methods (based on data)	Retrospective cohort/database	Ireland	2000-04	62	53
Kipnis 2016^49^	AAM	Using statistical methods (based on data)	Retrospective cohort/database	US	2010-13	65	46
Kirkland 2013^50^	—	Using statistical methods (based on data)	Other	US	2008-09	72	62
Kwon 2018^51^	DEWS	Using statistical methods (based on data)	Retrospective cohort/database	South Korea	2010-17	57	52
Kyriacos 2014^52^	MEWS*	Based on clinical consensus	NA	South Africa	NA	NA	NA
Luis 2018^53^	Short NEWS	Using statistical methods (based on data)	Retrospective cohort/database	Portugal	2012	Missing	48
Moore 2017^54^	UVA	Using statistical methods (based on data)	Retrospective cohort/database	Gabon, Malawi, Sierra Leone, Tanzania, Uganda, and Zambia	2009-15	36	49
Nickel 2016^55^	NEWS and D-dimer	Using statistical methods (based on data)	Retrospective cohort/database	Denmark	2008-11	62	45
Perera 2011^56^	MEWS plus biochemical	Using statistical methods (based on data)	Prospective cohort	Sri Lanka	2009	49	48
Prytherch 2010^57^	ViEWS	Using statistical methods (based on data)	Retrospective cohort/database	UK	2006-08	68	48
Redfern 2018^58^	LDTEWS:NEWS	Using statistical methods (based on data)	Retrospective cohort/database	UK	2011-16	73	49
Silke 2010^59^	MARS	Using statistical methods (based on data)	Retrospective cohort/database	Ireland	2002-07	50	48
Tarassenko 2011^60^	CEWS	Using statistical methods (based on data)	Prospective cohort	UK and US	2004-08	60	57
Watkinson 2018^61^	mCEWS	Using statistical methods (based on data)	Retrospective cohort/database	UK	2014-15	63	51
Wheeler 2013^62^	TOTAL	Using statistical methods (based on data)	Prospective cohort	Malawi	2012	40	51

AAM=advanced alert monitor; APPROVE=accurate prediction of prolonged ventilation; CARM=computer aided risk of mortality; CART=cardiac arrest risk triage; CEWS=centile early warning score; DENWIS=Dutch early nurse worry indicator score; DEWS=deep learning-based early warning system; DTEWS=decision tree early warning score; eCART=electronic cardiac arrest risk triage; EDI=early deterioration indicator; EMR=electronic medical record; GMEWS=global modified early warning score; HOTEL=hypotension, oxygen saturation, temperature, ECG [electrocardiogram] abnormality, loss of independence; LDTEWS=laboratory decision tree early warning score; MARS=medical admissions risk system; MEWS=modified early warning score; mCEWS=manual centile early warning score; NA=not available; NEWS=national early warning score; PSS=physiological scoring system; SCS=simple clinical score; TOTAL=tachypnoea, oxygen saturation, temperature, alert and loss of independence; UVA=universal vital assessment; ViEWS=VitalPAC early warning score.

* Not the same as original MEWS.

Most of the 29 studies that were developed by statistical methods used data from retrospective cohorts (n=21, 72%), while 7 (24%) used data from prospectively collected cohort datasets. Data used to develop the models were collected between 2000 and 2017. Twelve of the 29 studies (41%) did not adequately describe their dataset, missing at least one of the following characteristics: average age, distribution of men and women, number of patients with and without the event, and number of observation sets with and without the event (a patient could contribute more than one set of observations).

Twenty three of the 29 studies (79%) used a prediction modelling approach (including regression modelling and machine learning methods). The remaining six studies used a variety of methods. Appendix table C gives further details.

#### Outcome measures and time horizons

**Fig 2:**
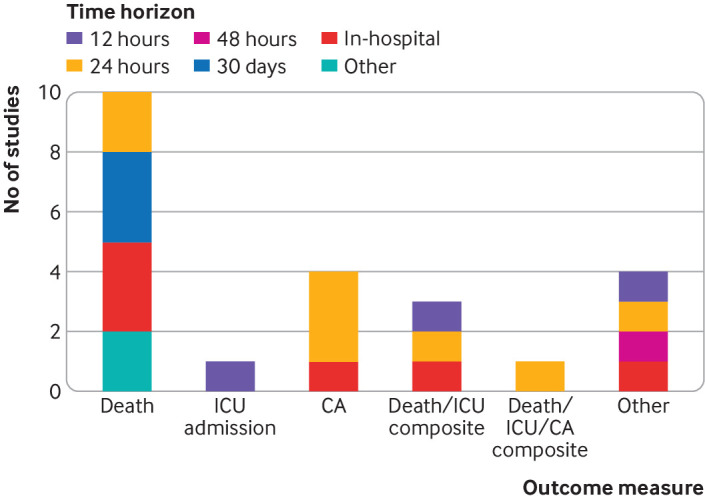
Summary of development outcomes and time horizons appearing in 23 studies that used regression modelling approach to develop early warning score. CA=cardiac arrest; ICU=intensive care unit

We observed a variety of primary outcome measures in the 23 development studies that used a prediction modelling approach (supplementary table A). Nearly all studies used death, intensive care unit admission, cardiac arrest, or a composite of these. The most common primary outcome measures were death (n=10, 44%) and cardiac arrest (n=4, 17%). A wide variety of prediction time horizons were also used; the most frequent was 24 hours (n=8, 35%). Other common horizons were 12 hours (n=3, 13%), 30 days (n=3, 13%), or in-hospital (n=6, 26%). Figure 2 shows a breakdown of outcomes and their time horizons.

#### Predictors

Twenty one of the 23 (91%) development studies that used a prediction modelling approach reported how many candidate predictors were considered for inclusion in the EWS, together reporting a median of 12 (range 4-45) predictors (supplementary table B). The median number of predictors included in the final model was seven (range 3-35). The most common approach for selecting variables for inclusion was backwards elimination (n=9/23, 39%). Six of the 23 models (26%) included all candidate variables, and three studies (13%) carried out univariable screening to reduce the initial number of candidate variables.

The most frequently included predictor in the 34 development studies was respiratory rate (n=30, 88%), followed by heart rate (n=28, 83%), oxygen saturation (n=24, 71%), temperature (n=24, 71%), systolic blood pressure (n=24, 71%), and level of consciousness (n=19, 56%). Thirteen models included age (38%) and three models included sex (9%).

#### Sample size (for 29 studies developed using statistical method)

The sample size in EWS studies can be complicated because there might be multiple observation sets for each patient or hospital admission. It was not always clear whether the reported sample size referred to the number of patients, hospital admissions, or observation sets (n=3; supplementary table C). The median patient or hospital admission sample size was 10 712 (range 242-649 418). Eleven of 29 articles (38%) used multiple observation sets for each patient, 15 (52%) used one observation set for each patient, and three (10%) were unclear. Of the 15 studies that used only one observation set for each patient, the first recorded observation was generally used (n=9, 60%).

The median number of events at the patient level was 396 (range 18-19 153) and at the observation set level was 284 (18-15 452). One article did not report the number of events at the patient level, and eight articles did not report the number of events at the observation set level. This difference in denominator explains how the median number of events can be greater at the patient level than at the observation set level.

The events per variable is a key marker of sample size adequacy in prediction modelling studies, and is defined as the number of events divided by the number of candidate predictor variables used. Twenty articles used a prediction modelling approach and provided sufficient information to calculate the patient level events per variable, with a median of 52 and a range from 1 to 1288. Fifteen studies provided enough information to calculate the observation set level events per variable, with a median of 17 and a range from 1 to 2693.

#### Statistical methods

Most of the articles that used statistical methods to develop an EWS mentioned missing data (n=25/29, 86%). Supplementary table C lists the methods for dealing with missing data; complete case analysis was the most common approach (n=10/25, 40%). None of the included studies used multiple imputation to handle missing data in the development of an EWS. Four articles mentioned missing data, but did not clearly state which method was used to handle them.

Most of the 23 models developed using a prediction modelling approach used logistic regression (n=15, 65%). Other methods included machine learning (n=4, 17%), Cox proportional hazards regression, multinomial logistic regression, discrete time logistic regression, and naïve Bayes classification combined with logistic regression (all n=1, 4%). The four machine learning studies used decision trees (n=2), artificial neural networks (n=1), or random forests (n=1).

For the handling of continuous predictors and use of interaction terms, all of the 23 prediction models included at least one continuous variable (supplementary table C). The most common approach for handling these variables was to categorise the variable before analysis (n=7, 30%). Other methods included splines (n=6, 26%), linear relations (n=4, 17%), and fractional polynomials (n=2, 9%). Four studies used other methods.

#### Model presentation

Nine of the 23 (39%) models developed by using a prediction modelling approach reported the complete regression formula, with all coefficients and either the intercept or baseline hazard (supplementary table E). Of the remaining models, seven (30%) did not report any coefficients, and seven (30%) reported the predictor coefficients but not the intercept or baseline hazard.

Thirteen of the studies (57%) reported enough information for us to calculate individualised risk predictions. Two articles (9%) reported the construction of risk groups. Ten articles (44%) created a simplified model, although only five described how this was done. These simplified models typically reduced the model coefficients to a points based scoring system, with no method of calculating predicted risks.

#### Apparent predictive performance

Twenty two studies assessed performance by using the same data that were used in the development of the model, thus assessing apparent performance (supplementary table F). Eighteen of these studies (82%) assessed discrimination with the C index, with values ranging from 0.69 to 0.96. Calibration was assessed for eight models (36%); seven used the Hosmer-Lemeshow goodness-of-fit test, and one used a calibration plot. Other performance metrics reported included sensitivity and specificity (n=8, 36%), and positive or negative predictive values (n=4, 18%). Eight studies presented receiver operating characteristic curves.

#### Internal validation

Supplementary table G shows reporting of internal validation in the 34 development studies. Nineteen models were internally validated. Note that two additional studies included an external validation of their new EWSs, but not an internal validation. Most studies split their data into development and validation data (n=13/19, 68%). Two articles used bootstrapping, and two used cross validation (both 11%). The remaining two assessed performance by using the derivation data combined with additional data (11%). All studies that assessed discrimination used the C index. Calibration was assessed in four studies, one using a calibration plot and three using the Hosmer-Lemeshow test. Sensitivity and specificity were reported in six studies and eight studies produced receiver operating characteristic curves.

### Studies describing external validation of EWS

We included 84 articles that externally validated an EWS (table 2). Twenty three of these also described the development of an EWS. Five developed an EWS and externally validated it in an external dataset, and 18 developed an EWS and externally validated a different EWS by using the development dataset.

**Table 2:** Design characteristics of 84 studies describing external validation of early warning score

Reference	Type of dataset	Country	Year	EWS validated	Mean age	Male (%)
Abbott 2016^63^	Prospective cohort	UK	2013	NEWS	63	48
Abbott 2015^64^	Prospective cohort	UK	2013	NEWS	61	46
Alvarez 2013^32^	Prospective cohort	US	2009-10	MEWS	51	54
Atmaca 2018^65^	Prospective cohort	Turkey	2014	NEWS	57	55
Badriyah 2014^33^	Retrospective cohort/database	UK	2006-08	NEWS	68	47
Bartkowiak 2019^66^	Retrospective cohort/database	US	2008-16	eCART, NEWS, MEWS	54	43
Beane 2018^67^	Retrospective cohort/database	Sri Lanka	2015	MEWS, NEWS, CART, ViEWS	43	41
Bleyer 2011^34^	Retrospective cohort/database	US	2008-09	NEWS, ViEWS	57	51
Brabrand 2017^68^	Retrospective cohort/database	Denmark	2012	NEWS, Worthing, Groarke, Goodacre	67	50
Brabrand 2018^69^	Retrospective cohort/database	Denmark	Missing	NEWS	74	49
Cei 2009^70^	Prospective cohort	Italy	2005-06	MEWS	79	44
Churpek 2017^71^	Retrospective cohort/database	US	2008-16	eCART, NEWS, MEWS	57	46
Churpek 2017^72^	Retrospective cohort/database	US	2008-16	NEWS, MEWS	57	48
Churpek 2013^73^	Retrospective cohort/database	US	2008-11	CEWS, MEWS, ViEWS, CART	55	44
Churpek 2014^36^	Retrospective cohort/database	US	2008-13	MEWS	60	40
Churpek 2012^74^	Other	US	2008-11	MEWS	59	52
Churpek 2012^8^	Retrospective cohort/database	US	2008-11	CART, MEWS	54	43
Churpek 2014^35^	Retrospective cohort/database	US	2008-11	ViEWS	54	43
Cooksley 2012^75^	Retrospective cohort/database	UK	2009-11	NEWS, MEWS	63	51
Cuthbertson 2010^38^	Prospective cohort	UK	2005	EWS, MEWS	65	51
De Meester 2013^76^	Prospective cohort	Belgium	2009-10	MEWS	59	60
DeVoe 2016^77^	Retrospective cohort/database	US	2007-13	MEWS	75	61
Douw 2017^78^	Retrospective cohort/database	Netherlands	2013-14	DENWIS	60	47
Duckitt 2007^39^	Prospective cohort	UK	2003-05	EWS	73	52
Dziadzko 2018^40^	Retrospective cohort/database	US	2017	APPROVE, MEWS, NEWS	56	33
Eccles 2014^79^	Retrospective cohort/database	UK	2012	NEWS	70	50
Escobar 2012^41^	Retrospective cohort/database	US	2006-09	MEWS	65	45
Fairclough 2009^80^	Prospective cohort	UK	2004-06	MEWS	73	43
Faisal 2018^42^	Retrospective cohort/database	UK	2014-15	CARM	68	48
Finlay 2014^81^	Retrospective cohort/database	US	2009-10	MEWS	65	NR
Forster 2018^82^	Retrospective cohort/database	UK	2015-17	NEWS	63	47
Garcea 2006^83^	Retrospective cohort/database	UK	2002-06	EWS	57	NR
Gardner 2006^84^	Prospective cohort	UK	2003	MEWS	59	50
Ghanem 2011^85^	Prospective cohort	Israel	2008-09	MEWS	75	52
Ghosh 2018^43^	Retrospective cohort/database	US	2012-13	MEWS, NEWS	59	NR
Green 2018^86^	Retrospective cohort/database	US	2008-13	MEWS, NEWS, eCART	62	41
Harrison 2006^45^	Retrospective cohort/database	Australia	2000	MEWS	NR	NR
Hodgson 2017^87^	Retrospective cohort/database	UK	2012-14	NEWS	74	NR
Hydes 2018^88^	Retrospective cohort/database	UK	2010-14	NEWS, EWS, MEWS, MEWS+age, Worthing	57	61
Jo 2016^89^	Retrospective cohort/database	South Korea	2013-14	NEWS	70	63
Kellett 2012^90^	Retrospective cohort/database	Canada	2005-11	ViEWS	63	49
Kellett 2016^91^	Prospective cohort	Canada	2005-16	ViEWS	65	49
Kim 2018^92^	Retrospective cohort/database	South Korea	2014-15	NEWS	65	70
Kim 2017^93^	Retrospective cohort/database	South Korea	2008-15	MEWS	61	65
Kipnis 2016^49^	Retrospective cohort/database	US	2010-13	eCART, NEWS	65	46
Kovacs 2016^94^	Retrospective cohort/database	UK	2011-13	NEWS	57	47
Kruisselbrink 2016^95^	Prospective cohort	Uganda	2013	MEWS	43	54
Kwon 2018^51^	Retrospective cohort/database	South Korea	2017	MEWS	58	50
LeLagadec 2020^96^	Retrospective case-control	Australia	2014-17	NEWS	73	53
Lee 2018^97^	Retrospective cohort/database	South Korea	2013-14	NEWS	62	58
Liljehult 2016^98^	Retrospective cohort/database	Denmark	2012	NEWS	72	50
Luis 2018^53^	Retrospective cohort/database	Portugal	2012	NEWS	NR	48
Moore 2017^54^	Retrospective cohort/database	Gabon, Malawi, Sierra Leone, Tanzania, Uganda, and Zambia	2009-15	MEWS	36	49
Mulligan 2010^99^	Prospective cohort	UK	2007	EWS	48	85
Öhman 2018^100^	Retrospective cohort/database	Denmark	2008-10	MARS	65	50
Opio 2013^101^	Retrospective cohort/database	Uganda	2012	ViEWS	45	42
Opio 2013^102^	Prospective cohort	Ireland	2011-13	TOTAL	64	53
Pedersen 2018^103^	Retrospective cohort/database	Denmark	2014	NEWS	74	42
Perera 2011^56^	Prospective cohort	Sri Lanka	2009	MEWS	49	48
Pimentel 2019^104^	Retrospective cohort/database	UK	2012-16	NEWS	68	48
Plate 2018^105^	Retrospective cohort/database	Netherlands	2014-16	ViEWS	61	65
Prytherch 2010^57^	Retrospective cohort/database	UK	2006-08	EWS, Goldhill, MEWS, MEWS+age, Worthing	68	48
Redfern 2018^106^	Retrospective cohort/database	UK	2010-16	NEWS	63	47
Redfern 2018^58^	Retrospective cohort/database	UK	2016	LDTEWS:NEWS, NEWS	73	50
Roberts 2017^107^	Retrospective cohort/database	Sweden	2014-15	NEWS	NR	60
Romero 2017^108^	Retrospective cohort/database	US	2011	GMEWS, Kirkland, MEWS, NEWS, ViEWS, Worthing	59	49
Romero 2014^109^	Retrospective cohort/database	US	2011	MEWS, GMEWS, Worthing, ViEWS, NEWS	59	49
Rylance 2009^110^	Prospective cohort	Tanzania	2005	MEWS	NR	34
Silke 2010^59^	Retrospective cohort/database	Ireland	2000-04	MARS	59	48
Smith 2008^111^	Retrospective cohort/database	UK	2006	EWS, Goldhill, MEWS, MEWS+age, Worthing	68	48
Smith 2013^112^	Retrospective cohort/database	UK	2006-08	NEWS, EWS, Goldhill, MEWS, MEWS+age, Worthing	68	47
Smith 2016^113^	Retrospective cohort/database	UK	2011-13	NEWS	62	48
Smith 2016^114^	Retrospective cohort/database	US	2014-15	NEWS	53	NR
Spagnolli 2017^115^	Prospective cohort	Italy	2013-15	NEWS	72	50
Stark 2015^116^	Retrospective cohort/database	US	2013-14	MEWS	62	65
Stræede 2014^117^	Retrospective cohort/database	Denmark	2008-09	SCS, HOTEL	62	52
Subbe 2001^118^	Retrospective cohort/database	UK	2000	MEWS, MEWS+age	63	45
Suppiah 2014^119^	Prospective cohort	UK	2010	MEWS	56	50
Tirkkonen 2014^120^	Prospective cohort	Finland	2010	NEWS	65	53
Tirotta2017^121^	Prospective cohort	Italy	2012	MEWS, TOTAL	73	50
Vaughn 2018^122^	Retrospective cohort/database	US	2011-15	MEWS	54	NR
VonLilienfeld-Toal 2007^123^	Retrospective cohort/database	Missing	2002-04	MEWS	40	51
Watkinson 2018^61^	Retrospective cohort/database	UK	2015-17	CART, CEWS, Goldhill, MEWS, MEWS+age, NEWS	68	49
Wheeler 2013^62^	Prospective cohort	Malawi	2012	MEWS, HOTEL	40	51

APPROVE=accurate prediction of prolonged ventilation; CARM=computer aided risk of mortality; CART=cardiac arrest risk triage; CEWS=centile early warning score; DENWIS=Dutch early nurse worry indicator score; eCART=electronic cardiac arrest risk triage; EWS=early warning score; GMEWS=global modified early warning score; HOTEL=hypotension, oxygen saturation, temperature, ECG [electrocardiogram] abnormality, loss of independence; LDTEWS=laboratory decision tree early warning score; MARS=medical admissions risk system; MEWS=modified early warning score; NEWS=national early warning score; NR=not reported; SCS=Simple clinical score; TOTAL=tachypnoea, oxygen saturation, temperature, alert and loss of independence; ViEWS=VitalPAC early warning score.

#### Models validated

**Fig 3:**
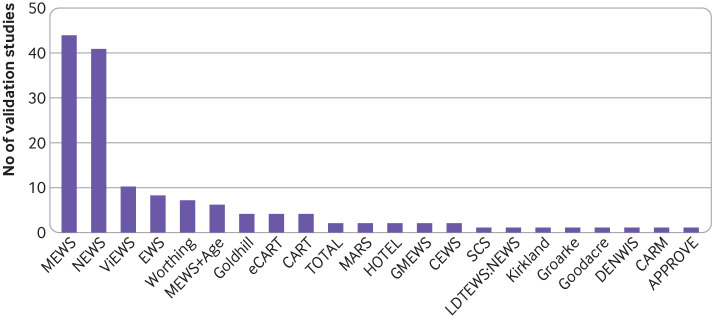
Frequency of external model validation by early warning score (EWS) in 84 included validation studies. Eight EWSs had never been externally validated. APPROVE=accurate prediction of prolonged ventilation; CARM=computer aided risk of mortality; CART=cardiac arrest risk triage; CEWS=centile early warning score; DENWIS=Dutch early nurse worry indicator score; eCART=electronic cardiac arrest risk triage; GMEWS=global modified early warning score; HOTEL=hypotension, oxygen saturation, temperature, ECG [electrocardiogram] abnormality, loss of independence; LDTEWS=laboratory decision tree early warning score; MARS=medical admissions risk system; MEWS=modified early warning score; NEWS=national early warning score; SCS=Simple clinical score; TOTAL=tachypnoea, oxygen saturation, temperature, alert and loss of independence; ViEWS=VitalPAC early warning score.

Twenty two models were validated across the 84 studies (fig 3). The modified early warning score^61^ was most frequently validated (n=43), followed by the national early warning score (NEWS)^44^ (n=40). The VitalPAC early warning score,^62^ on which NEWS was based, was validated 10 times, and the original EWS^5^ was validated eight times.

#### Study design

Most of the validation articles (n=58/84, 69%) used existing data to externally validate an EWS (table 2). Twenty five (30%) collected prospective data for external validation. The data used to validate the EWSs were all collected between 2000 and 2017. Thirty three of the 84 studies (39%) did not adequately describe their dataset, missing at least one of the following: average age, distribution of men and women, number of patients with or without the event, and number of observation sets with or without the event.

#### Outcome measures and horizon times

**Fig 4:**
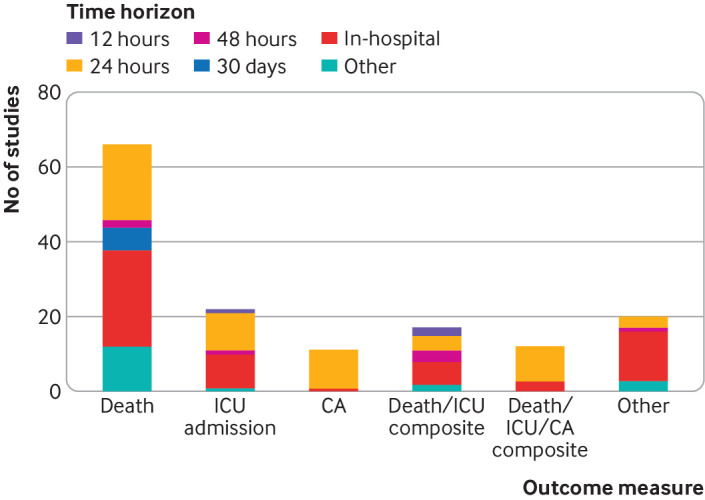
Summary of outcomes and time horizons used in 84 studies externally validating an early warning score. CA=cardiac arrest; ICU=intensive care unit

The models were validated against a range of outcomes (fig 4, supplementary table H). The most frequent was death, which was included in 66 of 84 articles (79%), followed by unanticipated admission to intensive care (n=22, 26%), and a composite of death and unanticipated admission to intensive care (n=17, 20%). A variety of prediction horizons were used. In-hospital (that is, the remainder of the hospital stay) was the most frequently used time point (n=58, 69%), followed by 24 hours (n=56, 67%). Figure 4 shows all outcome and time horizon combinations; in-hospital death was the most commonly validated endpoint (n=26, 31%).

#### Sample size

Supplementary table I shows reported information on the sample size used in each external validation. The number of patients and observation sets could not be identified in eight studies, and the number of event patients and event observation sets could not be identified in 25 studies. For studies that did report these data, the median number of patients included in the validation articles was 2806 (range 43 649 418) and the median number of observation sets was 3160 (range 43-48 723 248). The median number of event patients was 126, ranging from 6 to 19 153.

Multiple observation sets were used for each patient in 23 of 84 articles (27%), while one observation set for each patient was used in 55 articles (66%). The remaining six studies did not clearly report whether multiple observation sets had been used. Most of the studies that used a single observation set for each patient used the first observation set (n=41/55, 75%).

#### Statistical methods

Sixty three of the 84 validation articles (75%) mentioned missing data (supplementary table J). The most common approach for dealing with missing data was complete case analysis (n=36, 57%), followed by using the last observation carried forward (n=8, 13%). For seven studies the method was unclear (11%). Two articles reported having no missing data (3%). One article used multiple imputation (2%).

#### Predictive performance

Sixty nine of the 84 validation studies (82%) assessed model discrimination (supplementary table K). All of these studies used the C index, with values ranging from 0.55 to 0.96. Model calibration was assessed in 15 studies, most commonly by using the Hosmer-Lemeshow test (n=11, 73%). Calibration plots were presented in four studies (27%). Other commonly reported performance metrics included sensitivity and specificity (n=49, 58%), and positive or negative predictive values (n=31, 37%). Overall performance metrics, such as the Brier score and R^2^, were not reported in any of the studies.

Because of the heterogeneity of outcomes and time horizons used in the validation studies, and the relative lack of head-to-head comparisons, we did not quantitatively synthesise performance metrics for specific EWSs.

#### PROBAST risk of bias assessment

**Fig 5:**
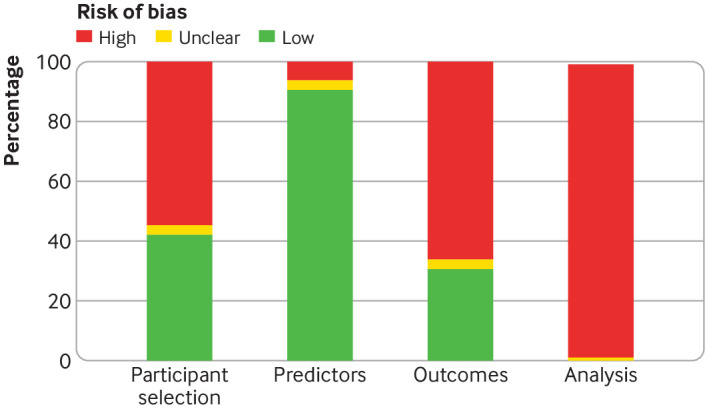
Summary of risk of bias in four domains of 95 studies developing or validating an early warning score, assessed using PROBAST (prediction model risk of bias assessment tool)

We assessed risk of bias for each study, focusing on participant selection, predictors, outcomes, and analysis (fig 5). Participant selection was at low risk of bias in 42% of studies and at high risk of bias in 55%. For the remaining studies, risk of bias was unclear. Predictors were at low risk of bias in 91% of the studies, and at high risk in 5%. Outcomes were at low risk of bias in 31% of studies, and at high risk in 66%. The analysis methods were at high risk of bias in all but two studies (98%).

## Discussion

Our review of 95 published studies found poor methods and inadequate reporting in most studies that developed or validated EWSs. Problems were observed across all aspects of study design and analysis. We found that handling of statistical issues, such as missing data and regression modelling approaches, was inadequate. Few studies assessed calibration, an essential aspect of model performance, and no study assessed clinical utility using net benefit approaches.^124^ Many studies also failed to report important details, such as sample size, number of events, population characteristics, and details of statistical methods. Several studies failed to report the full model, preventing (independent) external validation or implementation of the model in practice.

EWSs developed using inadequate methods will probably result in poorly performing scoring systems that fail to predict deterioration.^125^ Poor methods in external validation studies could lead to implementation of inferior scoring systems, with false reassurances about their predictive ability and generalisability. These reports could explain why recent systematic reviews have found little evidence of any clinical effectiveness of EWSs.^24^ ^126^ Although formal assessment of the methods and reporting quality in EWSs is needed, some reviews have found that studies describing the development or validation of an EWS were low quality, used poor statistical methods, and were at high risk of bias.^23^ ^24^

We assessed risk of bias by using PROBAST^30^ and found that most of the included studies were at risk of bias owing to participant selection, outcome definitions, and statistical analysis. The only domain for which most of the studies were at low risk of bias was predictor selection. Overall, all studies were at high risk of bias.

Our study included more external validation studies than development studies (11 development studies, 61 validation studies, and 23 studies that both developed and validated a model), which differs from reviews conducted in other clinical areas.^19^ ^21^ Our eligibility criteria might partly explain this difference. We stipulated that a development study should not be included if a model is developed for a specific subpopulation (eg, patients with respiratory disease), but an external validation study could be included if an EWS developed for a general population is evaluated in a specific subpopulation. A relatively large number of studies reported the use of prospective data (24% development studies and 30% validation studies). Because the data required for development and validation of EWSs are commonly collected routinely, some authors might be reporting a prospective decision to use future routinely acquired data rather than the implementation of a prospective data acquisition process.

EWSs have historically been implemented as part of bedside paper observation charts. Because the scores were calculated manually, simple scoring systems were necessary. These systems often relied on assigning points to each vital sign, typically three points, and summing the points to get a total score. However, these systems make the unlikely assumption that each vital sign has the same predictive value.^8^ The total score has little meaning, and no obvious correspondence to an absolute risk of an event exists.

Electronic health records are increasingly being used to record vital signs and calculate EWSs.^127^ These records allow more sophisticated EWSs to be implemented that make full use of available data and can be integrated into the clinical workflow of healthcare providers. Because the adoption of digital vital signs charting inevitably leads to further research, it is important that this research is of the highest quality, particularly when interest has surged in using machine learning or artificial intelligence. The results of our review suggest recommendations for future research (box 2).

Box 2 **Summary of recommendations for future practice**

**Provide key details of analysis population**

We suggest that articles report population demographics (eg, age and sex), source of data (country, hospital, and ward), number of patients with and without the event of interest, and number of observation sets with and without the event of interest.

**Use large enough sample size**

The sample size should be sufficient to robustly answer the question. For model development studies we recommend performing a sample size calculation specific to the context. For external validation studies we suggest including at least 100 event patients.

**Describe amount of missing data and use statistical methods to account for missing data**

Describe the frequency of missing data for each predictor and outcome. We recommend multiple imputation is the best practice approach for accounting for missing data in the analysis.

**Carefully consider outcome measures and time horizons**

Use an outcome measure that is clinically meaningful (that is, an outcome measure that can be prevented by appropriate treatment), and a time horizon in which deterioration can reasonably be expected to occur, and thus be predicted, which is probably a few days at most.

**Use best practice statistical methods and report full model**

If using a regression modelling approach to develop a new EWS, studies should allow for nonlinear predictor-outcome relations (eg, fractional polynomials) and avoid categorising predictors before analysis. Predictor interaction terms and competing risk approaches should be considered if appropriate. Newly developed models should always be fully described to allow independent evaluation and implementation.

**Always carry out internal validation of new models**

Internal validation is an important way of assessing how optimistic newly developed models might be. Split sample validation should be avoided and bootstrapping should be used.

**Test all aspects of model performance**

Assess both calibration and discrimination of EWSs. We also recommend using decision curve analysis to evaluate clinical utility.

### Recommendations for future research practice

Many of the recommendations are covered by the TRIPOD (transparent reporting of a multivariable prediction model for individual prognosis or diagnosis) statement, which is a reporting guideline for studies developing or validating prognostic (or diagnostic) models.

#### Describe the data

We found that datasets were often not described in sufficient detail to understand in whom the model was intended for use or in whom the model was evaluated. These details are crucial when interpreting an article that describes an EWS. We recommend that several critical factors should be reported by all studies: number of patients with and without the event of interest; whether multiple observation sets are used per patient—if so, the total number of observation sets with and without an associated event; data source (eg, country, hospital, and wards); patient characteristics (eg, age, sex, and admission method).

#### Use sufficiently large sample size

Although many of the studies in our review used a large sample acquired from electronic health records, some used a sample that was too small. For example, a quarter of model development studies had fewer than six events for each variable at the patient level, and four at the observation level. A quarter of external validation studies included fewer than 460 patients, and a quarter of studies included fewer than 35 event patients. As the outcomes used in EWS studies are usually rare (~1-2%), and the number of events is a critical factor, large sample sizes are often necessary. Guidance suggests that external validation studies require a minimum of 100 event patients, and preferably more than 200.^128^ Therefore, with their low event rates, EWS studies require data from many thousands of patients. Although defining the necessary sample size for model development studies is more complex, new guidance is available, which should be considered before embarking on new EWS studies.^129^ ^130^ Data driven variable selection methods increase the chance of overfitting and therefore should be avoided if possible.

#### Account for missing data

Most of the included studies mentioned missing data (86% of development studies and 75% of validation studies), although most of these studies used a complete case analysis to deal with missing data. Data are usually not missing at random, but are using missing selectively, for example based on patient characteristics or illness severity. Therefore, excluding records with incompletely observed predictor or outcome data can result in serious bias^131^ ^132^; for example, by inflating associations between predictors and outcomes.

We recommend that every study should describe how missing data were handled (for example, using complete case analysis, single imputation, or multiple imputation). The studies should also describe the amount of missing data overall, and for each predictor variable and outcome. We recommend that complete case analyses be avoided. Instead imputation approaches should be considered, with missing data imputed based on other known information. These approaches are now easy to implement in all standard software packages. Multiple imputation is widely regarded as the best approach.^133^ ^134^ ^135^ This method allows the uncertainty about missing data to be accounted for by creating multiple imputed datasets, then appropriately combining the results from each dataset. Before implementing multiple imputation the likely missing data mechanism should be thoughtfully considered. If imputation is appropriate, the setup of the imputation model should also be carefully considered (eg, the handling of categorical and skewed variables), and fully reported.^136^

#### Use appropriate outcome measures and time horizons

The included studies used a variety of outcome measures and time horizons to develop and validate EWSs. Both development and validation studies frequently used death and unanticipated intensive care unit admission, along with a variety of composite outcomes that included these outcomes. Some debate exists about which outcome measure is most appropriate.^27^

We found that 39% of development studies and 52% of validation studies included a time horizon that was either in-hospital or 30 days. These long term horizons will not lead to models that give early warning of deterioration.^24^ Instead the resulting models will identify generally unwell patients who are more likely to die or be admitted to the intensive care unit. We recommend that the time horizon should be limited to a few days at most, as any signs of deterioration linked to an observed outcome will probably not be seen for longer than this period.

#### Use best practice statistical approaches and report the full model

We observed that several of the articles reported regression modelling approaches that were methodologically weak. Nonlinear relations between predictors and outcomes were only included in 23% of development studies. However, this is an area of research in which such relations might readily exist. For example, both low and high respiratory rates can indicate increased risk. Similarly, interactions between predictors were only considered in 22% of studies. Models to predict the individual outcomes of intensive care unit admission or cardiac arrest were relatively frequent, but few studies accounted for death as a competing risk (intensive care unit admission or cardiac arrest not being possible if death has occurred). Failure to account for death as a competing risk could lead to a biased model, and inaccurate model predictions.^137^ We recommend that future work accounts for competing risks in model development using Fine and Gray, cause specific hazards, or absolute risk regression^138^ ^139^ ^140^ ^141^ rather than logistic and Cox proportional hazards regression models. External validation of such models also requires that the potential of competing risks is taken into account.^142^ ^143^ We also observed that the full model (all regression coefficients and either an intercept or baseline survival) was poorly reported, with only 39% of studies reporting sufficient information to allow independent validation or implementation.

We recommend that future development studies use best practice statistical methods, including examining plausible interaction terms (which should be chosen a priori and not data driven), examining nonlinear relations, avoiding univariable selection methods, and reporting all regression modelling coefficients. The methods used should be fully described in the publication and follow the recommendations laid out in the TRIPOD statement.^17^

#### Use internal validation for new models

The apparent performance of a newly developed model on its development data is likely to be optimistic, and better than its performance when applied to external data. This optimism can be driven by a small sample size, many predictors, or categorisation of continuous variables. Internal validation quantifies the optimism and adjusts the apparent performance, and can be used to shrink the regression coefficients.^144^ ^145^ Although many studies randomly split their dataset into two parts, one for model development and one for validation, this approach is weak and inefficient.^144^ We found that 16 of our 34 articles included development studies that internally validated their EWS. However, 11 used a split sample approach.

Cross validation and bootstrapping are two preferred approaches for internal validation.^146^ These methods use the entire dataset to both develop and validate the model. They also correct for overfitting in the model performance. We prefer bootstrapping because it can account for the optimism associated with the full model building process (eg, variable selection methods), and it can also provide a mechanism to shrink the regression coefficients to compensate for overfitting.^144^ ^147^

We recommend that new EWSs be internally validated, using bootstrapping if possible. However, we recognise that large datasets are becoming ever more present. In this context bootstrapping can be time consuming and less worthwhile when large datasets are used because overfitting in these instances is less likely. We recommend a form of the split sample approach is carried out with large datasets, where the dataset is not split randomly, but according to time, location, or centre.^29^

#### Assess all aspects of model performance

Two key aspects characterise the performance of a prediction model, discrimination and calibration.^17^ ^148^ Discrimination refers to a prediction model’s ability to differentiate between those who develop an outcome and those who do not. A model should predict higher risks for those who develop the outcome. Calibration reflects the level of agreement between observed outcomes and the model predictions. In development studies the main emphasis will be on discrimination because the model will, by definition, be well calibrated. However, in external validation studies, both discrimination and calibration are important. Most of our included studies assessed model performance by using the C index, which has been observed in other prediction model reviews.^18^ ^21^ ^149^ ^150^ For example, although 82% of external validation articles reported a measure of discrimination, only 18% reported an assessment of calibration. Those that assessed calibration used weak methods that are not recommended. Only four articles (5%) presented the preferred approach to assess calibration, the calibration plot.

We recommend that both discrimination and calibration be assessed in external validation studies, in line with TRIPOD recommendations. Calibration should be assessed with a plot that compares predicted and observed risks, with a smoothed curve plotted using LOESS (locally estimated scatterplot smoothing) or similar methods, such as fractional polynomials or restricted cubic splines.^151^ Other metrics of calibration such as the intercept and slope should also be reported.^152^ Many EWSs are currently based on an integer scoring system. For example, NEWS ranges from 0 to 20 points. Calibration of an integer scoring system cannot be assessed because it relies on the model producing predicted probabilities. Overall performance measures, which combine discrimination and calibration, should also be considered, such as R^2^ and the Brier score.^152^ The more clinically meaningful decision curve analysis (or net benefit) approach is also recommended.^124^

### Weaknesses of the study

We assessed 34 development studies, which is perhaps fewer than expected compared with previous systematic reviews.^111^ We excluded several existing EWSs that have not been published in peer reviewed academic journals. However, we anticipate that the methods underlying these excluded EWSs will be of a similar standard, and possibly even worse, than those included in our review.

Our eligibility criteria state that external validation studies would only be included if the development study was also included. However, we chose to make an exception for studies that described external validation of Morgan’s original 1997 EWS, and Subbe’s modified early warning score. Otherwise this eligibility criterion excluded few articles.

Some other details that were not collected could be of interest for future investigation. For example, research could include the rationale for the choice of outcome measure and the prediction time horizon, and whether EWSs have been developed or validated by using and accounting for multicentre or clustered data.^153^

### Strengths of the study

This systematic review formally assessed the methods and reporting standards in EWS studies. We performed a thorough assessment of important aspects of development and validation based on the CHARMS checklist,^29^ and other important subject specific items. We also assessed risk of bias using the PROBAST tool.^30^

### Conclusion

We included 95 articles in our review that developed or externally validated EWSs. We found many methodological and reporting shortcomings. Therefore, EWSs in common use could perform more poorly than reported, with potentially detrimental effects on patient care. Clinical responses to elevated scores have major workload impacts,^154^ and the weaknesses of the EWSs affect the resulting workload. Therefore, healthcare professionals and policy makers need to be aware of these weaknesses when recommending particular response strategies.

Our study does not seek to recommend a particular EWS, however NEWS is currently mandated for use throughout the National Health Service in the UK.^155^ This system was developed by clinical consensus rather than by applying statistical methods, which is the usual method for developing prediction models. Claims of extensive validation^12^ might be misleading because we found the underlying methodology of EWS validation studies to be generally poor. In reality, clinicians can have little knowledge of how such scores will perform in their clinical setting. Therefore, clinicians should be cautious about relying on these scores to identify clinical deterioration in patients.

The move towards electronic implementation of EWSs presents an opportunity to introduce better scoring systems, particularly with the increasing interest in modern model building approaches, such as machine learning and artificial intelligence. However, if methodological and reporting standards are not improved, this potential might never be achieved.

**What is already known on this topic**

Early warning scores are widely used in hospitals to identify clinical deterioration in patients, for example the modified early warning score and the national early warning score
Early warning scores are commonly implemented by using electronic systems
A systematic overview of studies developing and externally validating these systems has been lacking

**What this study adds**

An abundance of articles describe the development or validation of early warning scores
Poor methods and inadequate reporting were found in most studies, and all studies were at risk of bias
Methodological problems could result in scoring systems that perform poorly in clinical practice, which might have detrimental effects on patient care

## Web extra

Extra material supplied by authors

**Web appendix:** Supplementary appendix

## Notes

Contributors: SG, TB, JB, SK, PJW, and GSC designed the study. SG and SK developed the search strings. SG, JB, and PSV extracted the data. SG analysed the data and wrote the first draft of the manuscript. All authors revised the manuscript and approved the final version of the submitted manuscript. The corresponding author attests that all listed authors meet authorship criteria and that no others meeting the criteria have been omitted. SG acts as the guarantor.

Ethical approval: Not required.

Data sharing: No additional data available.

The lead authors affirm that the manuscript is an honest, accurate, and transparent account of the study being reported; that no important aspects of the study have been omitted; and that any discrepancies from the study as planned have been explained.

Dissemination to participants and related patient and public communities: There are no study participants to disseminate the results to. Patients and participants involved in the study design and interpretation will be sent a copy of the article. The authors intend to disseminate the study findings through media organisations so that the results will be available for the wider patient and public community.
