# Supplementary material for: Early warning scores for detecting deterioration in adult hospital patients: systematic review and critical appraisal of methodology
Source: BMJ. 2020 May 20;369:m1501. doi: 10.1136/bmj.m1501 (PMC7238890; doi:10.1136/bmj.m1501)
Supplement: Supplementary file 1 — Web appendix: Supplementary appendix [file gers053616.ww.pdf]

Supplementary Appendix

Table A: Model development outcome measures and time horizons used in the 23 studies that used a prediction modelling approach to develop an early warning score.

Reference	Outcome measure	Prediction horizon
Alvarez 2013 [32]	Rescusitation / death composite	24 hours
Badriyah 2014 [33]	Death / ICU / cardiac arrest composite	24 hours
Churpek 2012 [8]	Cardiac arrest	In-hospital
Churpek 2014 [35]	Cardiac arrest / ICU composite	24 hours
Churpek 2014 [36]	Cardiac arrest	24 hours
Churpek 2016 [37]	Death	24 hours
Duckitt 2007 [39]	Death	In-hospital
Dziadzko 2018 [40]	Death / medical ventilation composite	48 hours
Escobar 2012 [41]	ICU admission	12 hours
Faisal 2018 [42]	Death	In-hospital
Ghosh 2018 [43]	Death / ICU composite	In-hospital
Goldhill 2004 [44]	Death	30 days
Kellett 2006 [47]	Death	30 days
Kellett 2008 [48]	Death	24 hours
Kipnis 2016 [49]	Death / ICU composite	12 hours
Kirkland 2013 [50]	Death / ICU / rapid response trigger composite	12 hours
Kwon 2018 [51]	Cardiac arrest	24 hours
Luis 2018 [53]	Death / ICU composite	24 hours
Moore 2017 [54]	Death	In-hospital
Nickel 2016 [55]	Death	30 days
Perera 2011 [56]	Death / ICU / cardiac arrest composite	In-hospital
Silke 2010 [59]	Death	5 days
Wheeler 2013 [62]	Death	72 hours

ICU: Intensive care unit

Table B: Predictor variables considered as candidates and included in the final model, reported for the papers reporting the development of an early warning score.

Reference	Number of candidate predictors	Number of predictors in final model	Predictors included in the final model
Albert 2011 [31]	NA	6	SBP, RR, HR, temperature, oxygen therapy, urine output
Alvarez 2013 [32]	45	13	DBP, SpO2, age, temperature, pCO2, WBC, platelets, potassium, arterial blood gas order, ECG order, CT / MRI Order, floor assignment, MEWS
Badriyah 2014 [33]	7	7	SBP, RR, HR, SpO2, temperature, oxygen therapy, level of consciousness
Bleyer 2011 [34]	NA	8	SBP, DBP, RR, HR, SpO2, temperature, level of consciousness, mean arterial pressure
Churpek 2012 [8]	16	4	DBP, RR, HR, age
Churpek 2014 [35]	22	15	DBP, RR, HR, SpO2, temperature, age, oxygen therapy, level of consciousness, prior ICU admission, blood urea nitrogen, anion gap, hemoglobin, platelets, potassium, WBC
Churpek 2014 [36]	Missing	27	SBP, DBP, RR, HR, SpO2, temperature, age, level of consciousness, previous ICU admission, pulse pressure, sodium, potassium, bicarbonate, anion gap, BUN, creatinine, BUN-creatinine ratio, glucose, calcium, WBC, hemoglobin, platelets, protein, albumin, bilirubin, AST, alkaline phosphatase
Churpek 2016 [37]	12	12	SBP, DBP, RR, HR, SpO2, and trends of these vital signs
Cuthbertson 2010 [38]	NA	3	RR, HR, SpO2
Douw 2016 [9]	NA	10	EWS, change in breathing, change in circulation, rigors, change in mentation, agitation, pain, unexpected trajectory, patient indicates, nurse observation
Duckitt 2007 [39]	6	6	SBP, RR, HR, SpO2, temperature, level of consciousness
Dziadzko 2018 [40]	35	35	SBP, DBP, RR, HR, temperature, age, sex, oxygen therapy, weight, BMI, pulse pressure, shock index, anion gap, arterial PaO2, arterial pH, BUN / Creatinine ratio, hematocrit, hemoglobin, lactate, serum albumin, serum anion gap, serum bicarbonate, serum blood gas nitrogen, serum calcium, serum chloride, serum creatinine, serum glucose, serum potassium, serum sodium, serum total

			bilirubin, white blood count, vasopressor use, oxygen delivery device, richmond agitation-sedation scale
Escobar 2012 [41]	30	17	SBP, DBP, RR, HR, SpO2, temperature, level of consciousness, directive status, LAPS, COPS, COPS missing, LOS, time of day, blood urea nitrogen , lactate, WBC
Faisal 2018 [42]	19	19	SBP, DBP, RR, HR, SpO2, temperature, age, sex, oxygen therapy, level of consciousness, NEWS, albumin, creatinine, haemoglobin, potassium, sodium, urea, white cell count, AKI
Ghosh 2018 [43]	8	6	SBP, RR, HR, SpO2, temperature, age
Goldhill 2004 [44]	7	5	SBP, RR, HR, age, level of consciousness
Harrison 2006 [45]	NA	5	SBP, RR, HR, temperature, level of consciousness
Jones 2012 [46]	NA	7	SBP, RR, HR, SpO2, temperature, oxygen therapy, level of consciousness
Kellett 2006 [47]	Missing	15	SBP, RR, HR, SpO2, temperature, age, breathless on presentation, abnormal ECG, diabetes, coma, altered mental status, stroke, lack on independence, nursing home resident, spends time in bed
Kellett 2008 [48]	11	5	SBP, SpO2, temperature, abnormal ECG, loss of independence
Kipnis 2016 [49]	38	34	SBP, DBP, RR, HR, SpO2, temperature, age, level of consciousness, admission category, anion gap, bicarbonate, SBP instability, care order, time of day, glucose, hematocrit, lactate, LAPS, BUN, COPS, creatinine, ELOS, ELOSO*LAPS, HR instability, SpO2 instability, worst SpO2, RR instability, troponin missing, lactate, worst RR, season, sodium, troponin, WBC
Kirkland 2013 [50]	10	4	SpO2, Braden scale, shock index (HR / SBP)
Kwon 2018 [51]	4	4	SBP, RR, HR, temperature
Kyriacos 2014 [52]	NA	7	SBP, RR, HR, SpO2, temperature, level of consciousness, urine
Luis 2018 [53]	7	6	SBP, RR, HR, SpO2, oxygen therapy, level of consciousness
Moore 2017 [54]	13	7	SBP, RR, HR, SpO2, temperature, level of consciousness, HIV status

Nickel 2016 [55]	4	4	NEWS, age, alternation in alertness, D-dimer
Perera 2011 [56]	14	14	SBP, RR, HR, Temperature, age, sex, level of consciousness, MEWS, albumin, CRP, CRP / albumin ratio, WBC, platelets, haemoglobin
Prytherch 2010 [57]	NA	6	RR, HR, SpO2, temperature, oxygen therapy, level of consciousness
Redfern 2018 [58]	NA	14	SBP, RR, HR, SpO2, temperature, oxygen therapy, level of consciousness, haemoglobin, WCC, urea, creatinine, sodium, potassium, albumin
Silke 2010 [59]	11	9	RR, HR, temperature, age, mean arterial pressure, potassium, urea, haematocrit, WCC
Tarassenko 2011 [60]	NA	4	SBP, RR, HR, SpO2
Watkinson 2018 [61]	NA	7	SBP, RR, HR, SpO2, temperature, oxygen therapy, level of consciousness
Wheeler 2013 [62]	12	5	RR, SpO2, temperature, level of consciousness, loss of independence

AKI: Acute kidney injury, AST: Aspartate transaminase, BMI: Body mass index, BUN: Blood urea nitrogen, COPS: Comorbidity point score, CRP: C-reactive protein, CT: Computed tomography, DBP: Diastolic blood pressure, ECG: Electrocardiogram, ELOS: Elapsed length of stay, HIV: Human immunodeficiency virus, HR: Heart rate, ICU: Intensive care unit, LAPS: Laboratory-based Acute Physiology Score, LOS: Length of stay, MEWS: Modified early warning score, MRI: Magnetic resonance imaging, NA: Not applicable, NEWS: National early warning score, PaO2: Partial pressure of oxygen, pCO2: Partial pressure of carbon dioxide, RR: Respiratory rate, SBP: Systolic blood pressure, SpO2: Oxygen saturation, WBC: White blood cell

Table C: Details of the methods used by the 29 studies that developed an early warning score using a statistical approach.

Reference	Modelling approach	Variable selection approach	Handling of continuous variables	Was missing data referred to	Missing data approach
Alvarez 2013 [32]	Logistic	All significant in univariable	Splines	Yes	Other (missing category used)
Badriyah 2014 [33]	Tree	All	Categorised / dictomised	No	NA
Bleyer 2011 [34]	NA (Critical vital signs identified according to those that lead to a 5/10/20% risk of mortality)	NA	NA	Yes	Use of complete cases
Churpek 2012 [8]	Logistic	Backward	Splines	Yes	Other (LOCF)
Churpek 2014 [35]	Multinomial logistic	Backward	Linear	No	NA
Churpek 2014 [36]	Cox	Backward	Splines	Yes	Other (LOCF)
Churpek 2016 [37]	Logistic	All	Splines	Yes	Other (LOCF)
Cuthbertson 2010 [38]	NA (discriminant analysis)	NA	NA	Yes	Use of complete cases
Duckitt 2007 [39]	Logistic	All	Splines	Yes	Use of complete cases
Dziadzko 2018 [40]	Random forest	Unclear	Other (Forest)	Yes	Other (random forest)
Escobar 2012 [41]	Logistic	Other	Linear	Yes	Unclear
Faisal 2018 [42]	Logistic	All	Other (Univariable transformations)	Yes	Use of complete cases
Ghosh 2018 [43]	Naïve Bayes / Logistic	Other	Other (Naïve Bayes Classification)	Yes	Unclear

Goldhill 2004 [44]	Logistic	Backward	Categorised / dictomised	Yes	No missing data
Kellett 2006 [47]	Logistic	All significant in univariabl e	Categorised / dictomised	Yes	Use of complete case s
Kellett 2008 [48]	Logistic	Backward	Categorised / dictomised	Yes	Use of complete case s
Kipnis 2016 [49]	Other - Discrete time logistic regression	Backward	Fractional polynomials	Yes	Unclear
Kirkland 2013 [50]	Logistic	Backward	Splines	Yes	Other (LOCF)
Kwon 2018 [51]	ANN	All	Other (Neural Network)	Yes	Other (LOCF)
Luis 2018 [53]	Logistic	All significant in univariabl e	Linear	Yes	Use of complete cases
Moore 2017 [54]	Tree	Backward	Categorised / dictomised	Yes	Single Imputation
Nickel 2016 [55]	Logistic	Other	Categorised / dictomised	No	NA
Perera 2011 [56]	Logistic	All	Linear	Yes	Use of complete case s
Prytherch 2010 [57]	NA (trial and error)	NA	NA	Yes	Unclear
Redfern 2018 [58]	NA (combines two published scores as a function of time)	NA	NA	Yes	Other (LOCF)
Silke 2010 [59]	Logistic	Other	Fractional polynomials	Yes	Use of complete case s
Tarassenko 2011 [60]	NA (centiles of vital sign distributions)	NA	NA	No	NA
Watkinson 2018 [61]	NA (centiles of vital sign distributions)	NA	NA	Yes	Other (population mean)
Wheeler 2013 [62]	Logistic	Backward	Categorised / dictomised	Yes	Use of complete case s

NA: Not applicable

Table D: Sample size reported in the 34 articles describing the development of an early warning scorer.

Reference	Multiple observations per patient	If single, which one	No. patients	No. observations	No. event patients	No. event observations	Events patients per variable	Event observations per variable
Alvarez 2013 [32]	Yes	NA	3624	23127	Missing	298	Missing	7
Badriyah 2014 [33]	Yes	NA	35585	198755	3149	Missing	450	Missing
Bleyer 2011 [34]	Yes	NA	27722	191667	760	Missing	Missing	Missing
Churpek 2012 [8]	No	Other (max/min)	44607	44607	88	88	6	6
Churpek 2014 [35]	Unclear	NA	59301	Missing	2652	Missing	121	Missing
Churpek 2014 [36]	Unclear	NA	162068	Missing	264	Missing	Missing	Missing
Churpek 2016 [37]	No	Other (most recent or summary stats)	269999	269999	15452	15452	1288	1288
Cuthbertson 2010 [38]	No	Other (summary stats)	466	466	129	129	NA	NA
Duckitt 2007 [39]	No	First	3184	3184	270	270	45	45
Dziadzko 2018 [40]	Yes	NA	34387	Missing	958	Missing	27	Missing
Escobar 2012 [41]	Yes	NA	102422	1079062	3525	4036	118	135
Faisal 2018 [42]	No	First	30996	30996	1766	1766	93	93
Ghosh 2018 [43]	No	Other (unclear)	9265	9265	882	882	110	110
Goldhill 2004 [44]	No	First	433	433	26	26	4	4
Kellett 2006 [47]	No	First	6736	6736	316	316	Missing	Missing
Kellett 2008 [48]	No	First	6947	6947	40	40	4	4
Kipnis 2016 [49]	Yes	NA	649418	48723248	19153	Missing	504	Missing

Kirkland 2013 [50]	Yes	NA	267	Missing	68	Missing	7	Missing
Kwon 2018 [51]	Yes	NA	46725	2769324	396	10772	99	2693
Luis 2018 [53]	No	Other (highest EWS)	330	330	34	34	5	5
Moore 2017 [54]	Unclear	NA	5573	Missing	966	Missing	74	Missing
Nickel 2016 [55]	No	First	1201	1201	69	69	17	17
Perera 2011 [56]	No	First	242	242	18	18	1	1
Prytherch 2010 [57]	No	Last	35585	198755	3133	1999	NA	NA
Redfern 2018 [58]	Yes	NA	97933	2490529	NA	NA	NA	NA
Silke 2010 [59]	No	First	10712	10712	648	648	59	59
Tarassenko 2011 [60]	Yes	NA	863	64622	NA	NA	NA	NA
Watkinson 2018 [61]	Yes	NA	12153	301644	NA	NA	NA	NA
Wheeler 2013 [62]	No	First	302	302	51	51	4	4

NA: Not applicable

Table E: Presentation of model development by the 23 studies developing an early warning score using a prediction modelling approach.

Reference	Full regression formula shown	Predictor coefficients shown	Risk groups created	Simplified model created
Alvarez 2013 [32]	No	Yes	Yes	No
Badriyah 2014 [33]	No	No	No	Yes
Churpek 2012 [8]	No	Yes	No	No
Churpek 2014 [35]	No	Yes	No	No
Churpek 2014 [36]	No	Yes	No	No
Churpek 2016 [37]	No	No	No	No
Duckitt 2007 [39]	No	Yes	No	Yes
Dziadzko 2018 [40]	No	No	No	No
Escobar 2012 [41]	Yes	Yes	No	No
Faisal 2018 [42]	Yes	Yes	No	No
Ghosh 2018 [43]	No	No	No	No
Goldhill 2004 [44]	No	Yes	No	Yes
Kellett 2006 [47]	Yes	Yes	Yes	Yes
Kellett 2008 [48]	Yes	Yes	No	Yes
Kipnis 2016 [49]	Yes	No	No	No
Kirkland 2013 [50]	Yes	Yes	No	No
Kwon 2018 [51]	No	No	No	No
Luis 2018 [53]	Yes	Yes	No	Yes
Moore 2017 [54]	No	No	No	Yes
Nickel 2016 [55]	Yes	Yes	No	Yes
Perera 2011 [56]	No	Yes	No	Yes
Silke 2010 [59]	Yes	Yes	No	No

Wheeler 2013 [62]	Yes	Yes	No	Yes

Table F: Assessment of apparent performance in the 34 studies developing an early warning score.

Reference	Apparent performance assessed	Discrimination assessed	Calibration assessed	ROC curve presented
Albert 2011 [31]	Yes	No	No	No
Alvarez 2013 [32]	Yes	Yes	Yes	No
Badriyah 2014 [33]	Yes	Yes	No	No
Bleyer 2011 [34]	Yes	Yes	No	Yes
Churpek 2012 [8]	Yes	Yes	No	Yes
Churpek 2014 [35]	No	NA	NA	NA
Churpek 2014 [36]	No	NA	NA	NA
Churpek 2016 [37]	No	NA	NA	NA
Cuthbertson 2010 [38]	Yes	Yes	No	No
Douw 2016 [9]	Yes	Yes	No	No
Duckitt 2007 [39]	Yes	Yes	Yes	Yes
Dziadzko 2018 [40]	Yes	No	No	No
Escobar 2012 [41]	Yes	Yes	Yes	No
Faisal 2018 [42]	Yes	Yes	Yes	Yes
Ghosh 2018 [43]	Yes	Yes	No	Yes
Goldhill 2004 [44]	Yes	No	No	No
Harrison 2006 [45]	Yes	No	No	No
Jones 2012 [46]	No	NA	NA	NA
Kellett 2006 [47]	Yes	Yes	No	Yes
Kellett 2008 [48]	Yes	Yes	No	No
Kipnis 2016 [49]	No	NA	NA	NA
Kirkland 2013 [50]	Yes	Yes	No	No
Kwon 2018 [51]	No	NA	NA	NA
Kyriacos 2014 [52]	No	NA	NA	NA
Luis 2018 [53]	Yes	Yes	Yes	Yes
Moore 2017 [54]	No	NA	NA	NA
Nickel 2016 [55]	Yes	Yes	Yes	No
Perera 2011 [56]	Yes	Yes	No	No
Prytherch 2010 [57]	No	NA	NA	NA
Redfern 2018 [58]	No	NA	NA	NA
Silke 2010 [59]	Yes	Yes	Yes	Yes
Tarassenko 2011 [60]	No	NA	NA	NA
Watkinson 2018 [61]	No	NA	NA	NA

Wheeler 2013 [62]	Yes	Yes	Yes	No

ROC: Receiver operating characteristic

Table G: Reporting of internal validation in the 34 studies developing an early warning score.

Reference	Internal validation performed	Internal validation method	Discrimination assessed	Calibration assessed	ROC curve presented
Albert 2011 [31]	No	NA	NA	NA	NA
Alvarez 2013 [32]	Yes	Split sample	Yes	Yes	Yes
Badriyah 2014 [33]	No	NA	NA	NA	NA
Bleyer 2011 [34]	No	NA	NA	NA	NA
Churpek 2012 [8]	No	NA	NA	NA	NA
Churpek 2014 [35]	Yes	Cross-validation	Yes	No	No
Churpek 2014 [36]	Yes	Split sample	Yes	Yes	No
Churpek 2016 [37]	Yes	Split sample	Yes	No	No
Cuthbertson 2010 [38]	No	NA	NA	NA	NA
Douw 2016 [9]	No	NA	Na	NA	NA
Duckitt 2007 [39]	Yes	Other (additional sample combined with derivation)	Yes	No	No
Dziadzko 2018 [40]	Yes	Split sample	Yes	No	Yes
Escobar 2012 [41]	Yes	Split sample	Yes	No	No
Faisal 2018 [42]	Yes	Bootstrapping	Yes	Yes	No
Ghosh 2018 [43]	Yes	Split sample	Yes	No	Yes
Goldhill 2004 [44]	No	NA	NA	NA	NA
Harrison 2006 [45]	No	NA	NA	NA	NA
Jones 2012 [46]	No	NA	NA	NA	NA
Kellett 2006 [47]	Yes	Split sample	Yes	No	Yes
Kellett 2008 [48]	Yes	Split sample	Yes	Yes	No

Kipnis 2016 [49]	Yes	Split sample	Yes	No	Yes
Kirkland 2013 [50]	Yes	Split sample	Yes	No	Yes
Kwon 2018 [51]	Yes	Split sample	Yes	No	Yes
Kyriacos 2014 [52]	No	NA	NA	NA	NA
Luis 2018 [53]	No	NA	NA	NA	NA
Moore 2017 [54]	Yes	Cross-validation	Yes	No	No
Nickel 2016 [55]	No	NA	NA	NA	NA
Perera 2011 [56]	No	NA	NA	NA	NA
Prytherch 2010 [57]	Yes	Other (additional sample combined with derivation)	Yes	No	Yes
Redfern 2018 [58]	Yes	Split sample	Yes	No	Yes
Silke 2010 [59]	No	NA	NA	NA	NA
Tarassenko 2011 [60]	No	NA	NA	NA	NA
Watkinson 2018 [61]	Yes	Split samples	Yes	No	No
Wheeler 2013 [62]	Yes	Bootstrapping	Yes	No	No

NA: Not applicable

Table H: Outcome measures and time horizons used in the 84 studies externally validating an early warning score.

Reference	Outcomes and time horizons
Abbott 2016 [63]	48hr Composite death / ICU admission
Abbott 2015 [64]	48hr Composite death / ICU admission; Length of stay
Alvarez 2013 [32]	24hr Composite resuscitation event / death
Atmaca 2018 [65]	In-hospital ICU admission; 24hr, In-hospital death; Length of stay
Badriyah 2014 [33]	24hr Composite death / ICU admission / cardiac arrest; 24hr Death; 24hr ICU admission; 24hr Cardiac arrest
Bartkowiak 2019 [66]	In-hospital composite cardiac arrest / ICU admission / death
Beane 2018 [67]	24hr, In-hospital composite death / ICU admission / cardiac arrest / resuscitation
Bleyer 2011 [34]	In-hospital death
Brabrand 2017 [68]	24hr, In-hospital death
Brabrand 2018 [69]	30day death
Cei 2009 [70]	In-hospital death; In-hospital composite death / ICU admission; Length of stay
Churpek 2017 [71]	48hr Composite death / ICU admission
Churpek 2017 [72]	In-hospital death; In-hospital composite death / ICU admission
Churpek 2013 [73]	In-hospital composite death / ICU admission / cardiac arrest; In-hospital death; In-hospital ICU admission; In-hospital cardiac arrest
Churpek 2014 [36]	24hr Cardiac arrest; 24hr Composite death / ICU admission / cardiac arrest; 24hr Death; 24hr ICU admission
Churpek 2012 [74]	24hr Cardiac arrest
Churpek 2012 [8]	In-hospital ICU admission
Churpek 2014 [35]	24hr Cardiac arrest; 24hr ICU admission
Cooksley 2012 [75]	In-hospital ICU admission; In-hospital death
Cuthbertson 2010 [38]	24hr ICU admission
De Meester 2013 [76]	5day Composite death / ICU admission
DeVoe 2016 [77]	In-hospital death; Return of spontaneous circulation

Douw 2017 [78]	In-hospital composite ICU admission / death
Duckitt 2007 [39]	In-hospital death
Dziadzko 2018 [40]	48hr composite death / mechanical ventilation
Eccles 2014 [79]	30day Death
Escobar 2012 [41]	12hr ICU admission
Fairclough 2009 [80]	In-hospital death
Faisal 2018 [42]	In-hospital death
Finlay 2014 [81]	24hr Death
Forster 2018 [82]	24hr Death
Garcea 2006 [83]	In-hospital composite death / ICU admission / surgery; In-hospital death; Severity of pancreatitis
Gardner 2006 [84]	In-hospital ICU admission; In-hospital death
Ghanem 2011 [85]	24hr, 5day, 10day, 30day, 60day, In-hospital death
Ghosh 2018 [43]	In-hospital composite death / ICU admission
Green 2018 [86]	24hr Composite death / ICU admission / cardiac arrest
Harrison 2006 [45]	In-hospital death
Hodgson 2017 [87]	In-hospital death
Hydes 2018 [88]	24hr Composite death / ICU admission / cardiac arrest
Jo 2016 [89]	In-hospital death
Kellett 2012 [90]	48hr, 5day, 10day, 20day, 30day Death
Kellett 2016 [91]	24hr, 5day, 10day, 20day, 30day Death
Kim 2018 [92]	In-hospital composite death / ICU admission / RBC transfusion (>=5packs in 24hr); In-hospital death; In-hospital ICU admission; RBC transfusion (>=5packs in 24hrs)
Kim 2017 [93]	In-hospital ICU admission
Kipnis 2016 [49]	12hr, 24hr Composite death / ICU admission
Kovacs 2016 [94]	24hr Composite death / ICU admission / cardiac arrest; 24hr Death; 24hr ICU admission; 24hr Cardiac arrest
Kruisselbrink 2016 [95]	7day Death
Kwon 2018 [51]	24hr Cardiac arrest; 24hr Death
LeLagadec ~~2019~~ 2020[96]	72hr Composite cardiac arrest / death / ICU admission / MET call
Lee 2018 [97]	In-hospital death
Liljehult 2016 [98]	30day Death

Luis 2018 [53]	24hr Composite death / ICU admission
Moore 2017 [54]	In-hospital death
Mulligan 2010 [99]	In-hospital composite death / ICU admission / DNAR
Ohman 2018 [100]	5day, 7day Death
Opio 2013 [101]	24hr Death
Opio 2013 [102]	24hr Death
Pedersen 2018 [103]	48hr Death; 48hr ICU admission
Perera 2011 [56]	In-hospital composite death / ICU admission / cardiac arrest
Pimentel 2019 [104]	24hr Death; 24hr Cardiac arrest; 24hr ICU admission; 24hr Composite death / cardiac arrest / ICU admission
Plate 2018 [105]	12hr, 24hr Death; 12hr, 24hr ICU admission; 12hr, 24hr Composite death / ICU admission
Prytherch 2010 [57]	6, 12, 18, 24hr Death
Redfern 2018 [106]	24hr, In-hospital death; 24hr, In-hospital composite ICU admission / death; In-hospital composite 3day long ICU admission / death
Redfern 2018 [58]	24hr Composite death / ICU admission; 24hr Death; 24hr ICU admission
Roberts 2017 [107]	30day Death
Romero 2017 [108]	24hr Composite ICU admission / resuscitation call / RRT activation
Romero 2014 [109]	3, 8, 12, 24, 36hr Composite ICU admission / resuscitation call / RRT activation
Rylance 2009 [110]	In-hospital death
Silke 2010 [59]	5day, 7day Death
Smith 2008 [111]	In-hospital death
Smith 2013 [112]	24hr Composite death / ICU admission / cardiac arrest; 24hr Death; 24hr ICU admission; 24hr Cardiac arrest
Smith 2016 [113]	24hr Death; 24hr ICU admission; 24hr Cardiac arrest; 24hr Composite death / ICU admission / cardiac arrest
Smith 2016 [114]	In-hospital composite death / ICU admission / RRT activation / pulmonary thromboembolus (PTE) / postoperative sepsis / re-operation; Length of stay; 30day Readmission
Spagnolli 2017 [115]	72hr, In-hospital death; 72hr, In-hospital ICU admission; 72hr, In-hospital composite death / ICU admission
Stark 2015 [116]	In-hospital death
Straede 2014 [117]	24hr, 30day Death

Subbe 2001 [118]	60day Death; In-hospital ICU admission
Suppiah 2014 [119]	In-hospital poor outcome
Tirkkonen 2014 [120]	30day, 60day, 180day Death; Adverse Event
Tirotta 2017 [121]	In-hospital death
Vaughn 2018 [122]	In-hospital ICU admission; In-hospital death
VonLilienfeld-Toal 2007 [123]	In-hospital death
Watkinson 2018 [61]	24hr Death; 24hr Cardiac arrest; 24hr ICU admission; 24hr Composite death / cardiac arrest / ICU admission
Wheeler 2013 [62]	72hr Death

ICU: Intensive care unit, MET: Medical emergency team, RRT: Rapid response team

Table I: Reported sample size used in the 84 studies externally validating an early warning score.

Reference	Multiple observations per patient	If single, which one	No. patients	No. observations	No. event patients	No. event observations
Abbott 2016 [63]	No	First	322	322	15	15
Abbott 2015 [64]	No	First	453	453	16	16
Alvarez 2013 [32]	Yes	NA	3792	23847	287	Missing
Atmaca 2018 [65]	No	Other (cross-section in time)	104	104	21	21
Badriyah 2014 [33]	Yes	NA	35585	198755	3149	Missing
Bartkowiak 2019 [66]	No	Other (max EWS)	32537	32537	1243	1243
Beane 2018 [67]	No	Other (max EWS)	16386	16386	502	502
Bleyer 2011 [34]	Yes	NA	27722	191667	760	
Brabrand 2017 [68]	No	First	5784	5784	32	32
Brabrand 2018 [69]	No	First	570	570	66	66
Cei 2009 [70]	No	First	1107	1107	141	141
Churpek 2017 [71]	No	Other (max EWS)	53849	53849	10527	10527
Churpek 2017 [72]	No	Other (max EWS)	12154	12154	729	729
Churpek 2013 [73]	Yes	NA	59643	Missing	3055	Missing
Churpek 2014 [36]	Unclear	NA	107911	Missing	160	Missing
Churpek 2012 [74]	No	First	440	440	88	88
Churpek 2012 [8]	No	Other (max EWS)	47339	47339	2820	2820
Churpek 2014 [35]	Unclear	NA	59301	Missing	2652	Missing
Cooksley 2012 [75]	No	First	840	840	157	157
Cuthbertson 2010 [38]	No	Other (summary stats)	466	466	129	129

De Meester 2013 [76]	No	First	509	509	18	18
DeVoe 2016 [77]	No	First	417	417	86	86
Douw 2017 [78]	No	First	3522	3522	97	97
Duckitt 2007 [39]	No	First	3184	3184	270	270
Dziadzko 2018 [40]	Yes		2258	Missing	35	Missing
Eccles 2014 [79]	No	First	196	196	23	23
Escobar 2012 [41]	Yes	NA	102422	1079062	3525	Missing
Fairclough 2009 [80]	No	First	300	300	Missing	Missing
Faisal 2018 [42]	No	First	26247	26247	1703	1703
Finlay 2014 [81]	Yes	NA	32472	1794910	617	Missing
Forster 2018 [82]	Yes	NA	8263	263840	467	Missing
Garcea 2006 [83]	No	First	110	110	21	21
Gardner 2006 [84]	No	First	334	334	16	16
Ghanem 2011 [85]	No	First	1072	1072	43	43
Ghosh 2018 [43]	No	Other (unclear)	9265	9265	882	882
Green 2018 [86]	Yes	NA	107868	Missing	5485	Missing
Harrison 2006 [45]	No	First	3160	3160	27	27
Hodgson 2017 [87]	No	First	942	942	123	123
Hydes 2018 [88]	Yes	NA	1112	39619	146	1001
Jo 2016 [89]	No	First	553	553	60	60
Kellett 2012 [90]	No	First	75419	75419	308	308
Kellett 2016 [91]	No	First	65417	65417	205	205
Kim 2018 [92]	No	First	530	530	59	59
Kim 2017 [93]	No	First	1219	1219	468	468
Kipnis 2016 [49]	Yes	NA	649418	48723248	19153	Missing
Kovacs 2016 [94]	Yes	NA	20626	517324	709	Missing
Kruisselbrink 2016 [95]	No	First	452	452	25	25
Kwon 2018 [51]	Yes	NA	5406	213369	23	1247
LeLagadec ~~2019~~ 2020 [96]	Yes	NA	331	5556	159	Missing

Lee 2018 [97]	No	First	1300	1300	43	43
Liljehult 2016 [98]	No	First	274	274	24	24
Luis 2018 [53]	No	Other (highest EWS)	330	330	34	34
Moore 2017 [54]	Unclear	NA	5573	Missing	966	Missing
Mulligan 2010 [99]	No	Other (max EWS)	71	71	17	17
Ohman 2018 [100]	No	First	3904	3904	67	67
Opio 2013 [101]	No	First	844	844	66	66
Opio 2013 [102]	Unclear	NA	2935	14591	40	Missing
Pedersen 2018 [103]	Yes	NA	11266	404093	1138	Missing
Perera 2011 [56]	No	First	242	242	18	18
Pimentel 2019 [104]	Yes	NA	251266	6229740	6871	Missing
Plate 2018 [105]	Yes	NA	1782	9113	Missing	272
Prytherch 2010 [57]	No	Last	35585	198755	3133	3133
Redfern 2018 [106]	No	Random	241966	5435344	6798	Missing
Redfern 2018 [58]	Yes	NA	16309	395404	Missing	Missing
Roberts 2017 [107]	No	First	358	358	249	249
Romero 2017 [108]	Yes	NA	34898	16780669	3332	Missing
Romero 2014 [109]	Yes	NA	34898	Missing	4747	Missing
Rylance 2009 [110]	No	First	709	709	77	77
Silke 2010 [59]	No	First	3597	3597	171	171
Smith 2008 [111]	No	First	9987	9987	835	835
Smith 2013 [112]	Yes	NA	35585	198755		1999
Smith 2016 [113]	Yes	NA	103998	2245778	2351	10767
Smith 2016 [114]	No	First	411	411	25	25
Spagnolli 2017 [115]	No	First	2677	2677	62	62
Stark 2015 [116]	No	First	62	62	35	35

Straede 2014 [117]	No	First	3046	3046	26	26
Subbe 2001 [118]	No	First	673	673	75	75
Suppiah 2014 [119]	No	Other (max/mean EWS)	142	142	22	22
Tirkkonen 2014 [120]	No	Random	615	615	26	26
Tirotta 2017 [121]	No	First	526	526	78	78
Vaughn 2018 [122]	Unclear	NA	504	Missing	49	Missing
VonLilienfeld-Toal 2007 [123]	No	Other (max EWS)	43	43	6	6
Watkinson 2018 [61]	Yes	NA	53385	1459422	3507	Missing
Wheeler 2013 [62]	No	First	302	302	51	51

NA: Not applicable

Table J: Approach to missing data reported by the 84 that externally validated an early warning score.

Reference	Missing data referred to	Missing data approach
Abbott 2016 [63]	Yes	Use of complete cases
Abbott 2015 [64]	Yes	Use of complete cases
Alvarez 2013 [32]	Yes	Other (missing category used)
Atmaca 2018 [65]	No	NA
Badriyah 2014 [33]	No	NA
Bartkowiak 2019 [66]	Yes	Other (LOCF)
Beane 2018 [67]	Yes	Single imputation
Bleyer 2011 [34]	Yes	Use of complete cases
Brabrand 2017 [68]	Yes	Use of complete cases
Brabrand 2018 [69]	Yes	Single imputation
Cei 2009 [70]	No	NA
Churpek 2017 [71]	No	NA
Churpek 2017 [72]	Yes	Other (LOCF)
Churpek 2013 [73]	Yes	Other (LOCF)
Churpek 2014 [36]	Yes	Other (LOCF)
Churpek 2012 [74]	Yes	Unclear
Churpek 2012 [8]	Yes	Other (LOCF)
Churpek 2014 [35]	No	NA
Cooksley 2012 [75]	Yes	Use of complete cases
Cuthbertson 2010 [38]	Yes	Use of complete cases
De Meester 2013 [76]	Yes	Use of complete cases
DeVoe 2016 [77]	Yes	Use of complete cases
Douw 2017 [78]	Yes	Other (LOCF)
Duckitt 2007 [39]	Yes	Use of complete cases
Dziadzko 2018 [40]	Yes	Other (LOCF)
Eccles 2014 [79]	No	NA
Escobar 2012 [41]	Yes	Unclear
Fairclough 2009 [80]	No	NA
Faisal 2018 [42]	Yes	Use of complete cases
Finlay 2014 [81]	No	NA
Forster 2018 [82]	Yes	Use of complete cases
Garcea 2006 [83]	No	NA
Gardner 2006 [84]	No	NA
Ghanem 2011 [85]	Yes	Unclear
Ghosh 2018 [43]	Yes	Unclear
Green 2018 [86]	Yes	Unclear
Harrison 2006 [45]	No	NA

Hodgson 2017 [87]	Yes	No missing data
Hydes 2018 [88]	No	NA
Jo 2016 [89]	Yes	Use of complete cases
Kellett 2012 [90]	Yes	Use of complete cases
Kellett 2016 [91]	Yes	Use of complete cases
Kim 2018 [92]	Yes	Use of complete cases
Kim 2017 [93]	No	NA
Kipnis 2016 [49]	Yes	Unclear
Kovacs 2016 [94]	Yes	Use of complete cases
Kruisselbrink 2016 [95]	Yes	Use of complete cases
Kwon 2018 [51]	Yes	LOCF
LeLagadec ~~2019~~ 2020 [96]	Yes	Other (assumed normal)
Lee 2018 [97]	Yes	Use of complete cases
Liljehult 2016 [98]	Yes	Use of complete cases
Luis 2018 [53]	Yes	Use of complete cases
Moore 2017 [54]	Yes	Single imputation
Mulligan 2010 [99]	Yes	Other (assumed normal)
Ohman 2018 [100]	Yes	Use of complete cases
Opio 2013 [101]	Yes	Use of complete cases
Opio 2013 [102]	No	NA
Pedersen 2018 [103]	Yes	Use of complete cases
Perera 2011 [56]	Yes	Use of complete cases
Pimentel 2019 [104]	Yes	Multiple imputation
Plate 2018 [105]	Yes	Use of complete cases
Prytherch 2010 [57]	Yes	Unclear
Redfern 2018 [106]	Yes	Use of complete cases
Redfern 2018 [58]	Yes	Other (LOCF)
Roberts 2017 [107]	Yes	Other (assumed normal)
Romero 2017 [108]	No	NA
Romero 2014 [109]	No	NA
Rylance 2009 [110]	Yes	Use of complete cases
Silke 2010 [59]	Yes	Use of complete cases
Smith 2008 [111]	No	NA
Smith 2013 [112]	No	NA
Smith 2016 [113]	Yes	Use of complete cases
Smith 2016 [114]	No	NA
Spagnolli 2017 [115]	Yes	No missing data
Stark 2015 [116]	No	NA
Straede 2014 [117]	Yes	Use of complete cases
Subbe 2001 [118]	Yes	Use of complete cases

Suppiah 2014 [119]	Yes	Use of complete cases
Tirkkonen 2014 [120]	Yes	Use of complete cases
Tirotta 2017 [121]	No	NA
Vaughn 2018 [122]	Yes	Use of complete cases
VonLilienfeld-Toal 2007 [123]	Yes	Other (assumed normal)
Watkinson 2018 [61]	Yes	Use of complete cases
Wheeler 2013 [62]	Yes	Use of complete cases

NA: Not applicable

Table K: Model performance reported by the 84 studies externally validating an early warning score.

Reference	Discrimination assessed	Calibration assessed	ROC curve presented
Abbott 2016 [63]	Yes	No	No
Abbott 2015 [64]	No	No	No
Alvarez 2013 [32]	Yes	No	Yes
Atmaca 2018 [65]	No	No	No
Badriyah 2014 [33]	Yes	No	No
Bartkowiak 2019 [66]	Yes	No	No
Beane 2018 [67]	Yes	No	No
Bleyer 2011 [34]	Yes	No	Yes
Brabrand 2017 [68]	Yes	No	Yes
Brabrand 2018 [69]	Yes	Yes	Yes
Cei 2009 [70]	No	No	No
Churpek 2017 [71]	Yes	No	No
Churpek 2017 [72]	Yes	No	No
Churpek 2013 [73]	Yes	No	No
Churpek 2014 [36]	Yes	Yes	No
Churpek 2012 [74]	Yes	No	No
Churpek 2012 [8]	Yes	No	Yes
Churpek 2014 [35]	Yes	No	No
Cooksley 2012 [75]	Yes	No	Yes
Cuthbertson 2010 [38]	Yes	No	No
De Meester 2013 [76]	Yes	No	No
DeVoe 2016 [77]	No	No	No
Douw 2017 [78]	Yes	No	No
Duckitt 2007 [39]	Yes	No	Yes
Dziadzko 2018 [40]	Yes	No	Yes
Eccles 2014 [79]	Yes	No	No
Escobar 2012 [41]	Yes	No	No
Fairclough 2009 [80]	No	No	No
Faisal 2018 [42]	Yes	Yes	Yes
Finlay 2014 [81]	Yes	No	Yes
Forster 2018 [82]	No	No	No
Garcea 2006 [83]	Yes	No	No
Gardner 2006 [84]	Yes	Yes	No
Ghanem 2011 [85]	Yes	Yes	Yes
Ghosh 2018 [43]	Yes	No	Yes
Green 2018 [86]	Yes	No	No
Harrison 2006 [45]	No	No	No

Hodgson 2017 [87]	Yes	Yes	Yes
Hydes 2018 [88]	Yes	No	No
Jo 2016 [89]	Yes	Yes	Yes
Kellett 2012 [90]	Yes	No	No
Kellett 2016 [91]	Yes	No	No
Kim 2018 [92]	Yes	Yes	Yes
Kim 2017 [93]	Yes	No	Yes
Kipnis 2016 [49]	Yes	No	Yes
Kovacs 2016 [94]	Yes	No	No
Kruisselbrink 2016 [95]	Yes	No	No
Kwon 2018 [51]	Yes	No	Yes
LeLagadec ~~2019~~ 2020 [96]	Yes	No	No
Lee 2018 [97]	Yes	No	No
Liljehult 2016 [98]	Yes	No	Yes
Luis 2018 [53]	Yes	No	Yes
Moore 2017 [54]	Yes	No	No
Mulligan 2010 [99]	Yes	No	Yes
Ohman 2018 [100]	Yes	Yes	Yes
Opio 2013 [101]	Yes	No	No
Opio 2013 [102]	Yes	No	No
Pedersen 2018 [103]	Yes	No	No
Perera 2011 [56]	Yes	No	No
Pimentel 2019 [104]	Yes	No	Yes
Plate 2018 [105]	Yes	Yes	No
Prytherch 2010 [57]	Yes	No	Yes
Redfern 2018 [106]	Yes	No	No
Redfern 2018 [58]	Yes	No	Yes
Roberts 2017 [107]	No	No	No
Romero 2017 [108]	No	No	No
Romero 2014 [109]	No	No	Yes
Rylance 2009 [110]	No	No	Yes
Silke 2010 [59]	Yes	Yes	Yes
Smith 2008 [111]	Yes	No	Yes
Smith 2013 [112]	Yes	No	No
Smith 2016 [113]	Yes	No	Yes
Smith 2016 [114]	Yes	No	Yes
Spagnolli 2017 [115]	No	No	No
Stark 2015 [116]	No	No	No
Straede 2014 [117]	Yes	Yes	Yes
Subbe 2001 [118]	Yes	No	Yes

Suppiah 2014 [119]	Yes	No	Yes
Tirkkonen 2014 [120]	No	No	No
Tirotta 2017 [121]	Yes	No	Yes
Vaughn 2018 [122]	Yes	No	No
VonLilienfeld-Toal 2007 [123]	No	No	Yes
Watkinson 2018 [61]	Yes	No	No
Wheeler 2013 [62]	Yes	Yes	Yes

ROC: Receiver operating characteristic

EMBASE search strategy

Database & Platform: Embase (OVID) 1974 to 2017 Week 35

Search date: 30 August 2017

1. ((National or VitalPAC or Modified or Centile or standard$) adj1 Early adj1 Warning adj1 Scor$).ti,ab.

2. (Reading adj1 Modified adj1 Early adj1 Warning adj1 Score).ti,ab.

3. (Cardiac adj1 Arrest adj1 Risk adj1 Triage).ti,ab.

4. (Assessment adj1 Score adj2 Sick adj1 patient adj1 Identification adj2 Step-up adj2 Treatment).ti,ab.

5. (Targeted adj1 Real adj1 Time adj1 Early adj1 Warning adj1 Score).ti,ab.

6. (Dutch adj1 Early adj1 Nurse adj1 Worry adj1 Indicator adj1 Score).ti,ab.

7. (Decision adj1 Tree adj1 Early adj1 Warning adj1 Score).ti,ab.

8. (Advanced adj1 Alert adj1 Monitor).ti,ab.

9. (Chronic adj1 Respiratory adj1 Early adj1 Warning adj1 Score).ti,ab.

10. (early adj1 warning adj1 (scor$ or system$)).ti,ab.

11. (track adj2 trigger adj2 (scor$ or system$)).ti,ab.

12. (physiological adj1 scoring adj1 system$).ti,ab.

13. (worry adj1 indicator adj1 scor$).ti,ab.

14. (physiological adj1 observation adj1 track adj2 trigger adj1 (scor$ or system$)).ti,ab.

15. (patient adj2 risk adj2 scoring adj1 system$).ti,ab.

16. (patient adj2 risk adj1 trigger adj1 scoring adj1 system).ti,ab.

17. (early adj1 detection adj2 patients adj2 risk).ti,ab.

18. Early Warning Score.kw.

19. Early Warning Scores.kw.

20. Early warning system.kw.

21. Early warning systems.kw.

22. Track-and-Trigger.kw.

23. "track and trigger".kw.

24. Early Warning Score/

25. Early Warning Scores/

26. National Early Warning Score/

27. Modified Early Warning Score/

28. "Track and Trigger System"/

29. OR/1-28

30. Vital Sign/

31. Scoring System/

32. Physiologic Monitoring/

33. (NEWS or ViEWS or CART or SEWS or CREWS or PAR or PART or PSS or AAM).ti,ab.

34. OR/30-33

35. ((early or risk or warn$ or alert$ or track$ or trigger) adj2 (scor$ or system or systems)).ti,ab.

36. 34 AND 35

37. (MEWS or R-MEWS or eCART or CEWS or TREWScore or DENWIS or DTEWS or DMEWS or POTTS or PAR-T or ViEWS-L).ti,ab.

38. (develop$ or design$ or creat$ or build$ or contruct$ or validat$).ti,ab.

39. Validation Study/

40. 38 or 39

41. 36 and 40

42. 37 and 40

43. 41 or 42

44. 29 or 43

45. ((child OR infant OR pediatrics) NOT adult).sh.

46. 44 NOT 45

CINAHL search strategy

Database & Platform: CINAHL (via EbscoHost)

Search date: 30 August 2017

1. TI ((National or VitalPAC or Modified or Centile or standard*) N1 Early N1 Warning N1 Scor*) OR AB ((National or VitalPAC or Modified or Centile or standard*) N1 Early N1 Warning N1 Scor*) OR IN ((National or VitalPAC or Modified or Centile or standard*) N1 Early N1 warning N1 Scor*)

2. TI (Reading N1 Modified N1 Early N1 Warning N1 Score) OR AB (Reading N1 Modified N1 Early N1 Warning N1 Score) OR IN (Reading N1 Modified N1 Early N1 Warning N1 Score)

3. TI (Cardiac N1 Arrest N1 Risk N1 Triage) OR AB (Cardiac N1 Arrest N1 Risk N1 Triage) OR IN (Cardiac N1 Arrest N1 Risk N1 Triage)

4. TI (Assessment N1 Score N2 Sick N1 patient N1 Identification N2 Step-up N2 Treatment) OR AB (Assessment N1 Score N2 Sick N1 patient N1 Identification N2 Step-up N2 Treatment) OR IN (Assessment N1 Score N2 Sick N1 patient N1 Identification N2 Step-up N2 Treatment)

5. TI (Targeted N1 Real N1 Time N1 Early N1 Warning N1 Score) OR AB (Targeted N1 Real N1 Time N1 Early N1 Warning N1 Score) OR IN (Targeted N1 Real N1 Time N1 Early N1 Warning N1 Score)

6. TI (Dutch N1 Early N1 Nurse N1 Worry N1 Indicator N1 Score) OR AB (Dutch N1 Early N1 Nurse N1 Worry N1 Indicator N1 Score) OR IN (Dutch N1 Early N1 Nurse N1 Worry N1 Indicator N1 Score)

7. TI (Decision N1 Tree N1 Early N1 Warning N1 Score) OR AB (Decision N1 Tree N1 Early N1 Warning N1 Score) OR IN (Decision N1 Tree N1 Early N1 Warning N1 Score)

8. TI (Advanced N1 Alert N1 Monitor) OR AB (Advanced N1 Alert N1 Monitor) OR IN (Advanced N1 Alert N1 Monitor)

9. TI (Chronic N1 Respiratory N1 Early N1 Warning N1 Score) OR AB (Chronic N1 Respiratory N1 Early N1 Warning N1 Score) OR IN (Chronic N1 Respiratory N1 Early N1 Warning N1 Score)

10. TI (early N1 warning N1 (scor* or system*)) OR AB (early N1 warning N1 (scor* or system*))

11. TI (track N2 trigger N2 (scor* or system*)) OR AB (track N2 trigger N2 (scor* or system*))

12. TI (physiological N1 scoring N1 system*) OR AB (physiological N1 scoring N1 system*)

13. TI (worry N1 indicator N1 scor*) OR AB (worry N1 indicator N1 scor*)

14. TI (physiological N1 observation N1 track N2 trigger N1 (scor* or system*)) OR AB (physiological N1 observation N1 track N2 trigger N1 (scor* or system*))

15. TI (patient N2 risk N2 scoring N1 system*) OR AB (patient N2 risk N2 scoring N1 system*)

16. TI (patient N2 risk N1 trigger N1 scoring N1 system) OR AB (patient N2 risk N1 trigger N1 scoring N1 system)

17. TI (early N1 detection N2 patients N2 risk) OR AB (early N1 detection N2 patients N2 risk)

18. IN "early warning"

19. IN "trigger"

20. S1 OR S2 OR S3 OR S4 OR S5 OR S6 OR S7 OR S8 OR S9 OR S10 OR S11 OR S12 OR S13 OR S14 OR S15 OR S16 OR S17 OR S18 OR S19

21. MH "Early intervention"

22. MH "Predictive Value of Tests"

23. MH "Monitoring, Physiologic"

24. MH "Nursing Assessment"

25. MH "Severity of Illness Indices" OR MJ "Severity of illness"

26. MH "Instrument validation"

27. MH "Vital signs"

28. TI (NEWS or ViEWS or CART or SEWS or CREWS or PAR or PART or PSS or AAM) OR AB (NEWS or ViEWS or CART or SEWS or CREWS or PAR or PART or PSS or AAM)

29. S21 OR S22 OR S23 OR S24 OR S25 OR S26 OR S27 OR S28

30. TI ((early or risk or warn* or alert* or track* or trigger) N2 (scor* or system or systems)) OR AB ((early or risk or warn* or alert* or track* or trigger) N2 (scor* or system or systems))

31. S29 AND S30

32. TI (MEWS or R-MEWS or eCART or CEWS or TREWScore or DENWIS or DTEWS or DMEWS or POTTS or PAR-T or ViEWS-L) OR AB (MEWS or R-MEWS or eCART or CEWS or TREWScore or DENWIS or DTEWS or DMEWS or POTTS or PAR-T or ViEWS-L)

33. TI (develop* or design* or creat* or build* or contruct* or validat*) OR AB (develop* or design* or creat* or build* or contruct* or validat*)

34. MH "Validation Studies"

35. S33 OR S34

36. S31 AND S35

37. S32 AND S35

38. S36 OR S37

39. S20 OR S38

40. (MH (Child OR Infant OR Pediatrics) OR DH (Child OR Infant OR Pediatrics) NOT MH Adult)

41. S39 NOT S40

PsycINFO search strategy

Database & Platform: PsycINFO (OVID) 1967 to August Week 3 2017

Search date: 30 August 2017

1. ((National or VitalPAC or Modified or Centile or standard$) adj1 Early adj1 Warning adj1 Scor$).ti,ab,id.

2. (Reading adj1 Modified adj1 Early adj1 Warning adj1 Score).ti,ab,id.

3. (Cardiac adj1 Arrest adj1 Risk adj1 Triage).ti,ab,id.

4. (Assessment adj1 Score adj2 Sick adj1 patient adj1 Identification adj2 Step-up adj2 Treatment).ti,ab,id.

5. (Targeted adj1 Real adj1 Time adj1 Early adj1 Warning adj1 Score).ti,ab,id.

6. (Dutch adj1 Early adj1 Nurse adj1 Worry adj1 Indicator adj1 Score).ti,ab,id.

7. (Decision adj1 Tree adj1 Early adj1 Warning adj1 score).ti,ab,id.

8. (Advanced adj1 Alert adj1 Monitor).ti,ab,id.

9. (Chronic adj1 Respiratory adj1 Early adj1 Warning adj1 Score).ti,ab,id.

10. (early adj1 warning adj1 (scor$ or system$)).ti,ab,id.

11. (track adj2 trigger adj2 (scor$ or system$)).ti,ab.

12. (physiological adj1 scoring adj1 system$).ti,ab.

13. (worry adj1 indicator adj1 scor$).ti,ab.

14. (physiological adj1 observation adj1 track adj2 trigger adj1 (scor$ or system$)).ti,ab.

15. (patient adj2 risk adj2 scoring adj1 system$).ti,ab.

16. (patient adj2 risk adj1 trigger adj1 scoring adj1 system).ti,ab.

17. (early adj1 detection adj2 patients adj2 risk).ti,ab.

18. "early warning".id.

19. "track and trigger".id.

20. "trigger scor$".id.

21. OR/1-20

22. Warnings/

23. Monitoring, Physiologic.mh.

24. Nursing Assessment.mh.

25. Vital Signs.mh.

26. Severity of Illness Index.mh.

27. Monitoring/

28. "Scoring (Testing)"/

29. "vital Signs".id.

30. "severity of illness".id.

31. (NEWS or ViEWS or CART or SEWS or CREWS or PAR or PART or PSS or AAM).ti,ab.

32. OR/22-31

33. ((early or risk or warn$ or alert$ or track$ or trigger) adj2 (scor$ or system or systems)).ti,ab.

34. 32 AND 33

35. (MEWS or R-MEWS or eCART or CEWS or TREWScore or DENWIS or DTEWS or DMEWS or POTTS or PAR-T or ViEWS-L).ti,ab.

36. (develop$ or design$ or creat$ or build$ or contruct$ or validat$).ti,ab.

37. 34 and 36

38. 35 and 36

39. 37 OR 38

40. 21 or 39

41. ((child OR infant OR pediatrics) NOT adult).mh,sh.

42. 40 NOT 41

MEDLINE search strategy

Database and Platform: Ovid MEDLINE(R) Epub Ahead of Print, In-Process & Other Non-Indexed Citations, Ovid MEDLINE(R) Daily and Ovid MEDLINE(R) 1946 to Present

Search date: 30 August 2017

1. ((National or VitalPAC or Modified or Centile or standard$) adj1 Early adj1 Warning adj1 Scor$).ti,ab.
2. (Reading adj1 Modified adj1 Early adj1 Warning adj1 Score).ti,ab.
3. (Cardiac adj1 Arrest adj1 Risk adj1 Triage).ti,ab.
4. (Assessment adj1 Score adj2 Sick adj1 patient adj1 Identification adj2 Step-up adj2 Treatment).ti,ab.
5. (Targeted adj1 Real adj1 Time adj1 Early adj1 Warning adj1 Score).ti,ab.
6. (Dutch adj1 Early adj1 Nurse adj1 Worry adj1 Indicator adj1 Score).ti,ab.
7. (Decision adj1 Tree adj1 Early adj1 Warning adj1 Score).ti,ab.
8. (Advanced adj1 Alert adj1 Monitor).ti,ab.
9. (Chronic adj1 Respiratory adj1 Early adj1 Warning adj1 Score).ti,ab.
10. (early adj1 warning adj1 (scor$ or system$)).ti,ab.
11. (track adj2 trigger adj2 (scor$ or system$)).ti,ab.
12. (physiological adj1 scoring adj1 system$).ti,ab.
13. (worry adj1 indicator adj1 scor$).ti,ab.
14. (physiological adj1 observation adj1 track adj2 trigger adj1 (scor$ or system$)).ti,ab.
15. (patient adj2 risk adj2 scoring adj1 system$).ti,ab.
16. (patient adj2 risk adj1 trigger adj1 scoring adj1 system).ti,ab.
17. (early adj1 detection adj2 patients adj2 risk).ti,ab.
18. Early Warning Score.kw.
19. Early Warning Scores.kw.
20. Early Warning System.kw.
21. Early Warning Systems.kw.

22. Track-and-Trigger.kw.

23. "track and trigger".kw.

24. or/1-23

25. Predictive Value of Tests/

26. Monitoring, Physiologic/

27. Nursing Assessment/mt [Methods]

28. Nursing Assessment/st

29. Nursing Assessment/sn

30. Severity of Illness Index/

31. Health Status Indicators/

32. Point-of-Care-Systems/

33. (NEWS or ViEWS or CART or SEWS or CREWS or PAR or PART or PSS or AAM).ti,ab.

34. OR/25-33

35. ((early or risk or warn$ or alert$ or track$ or trigger) adj2 (scor$ or system or systems)).ti,ab.

36. 34 AND 35

37. (MEWS or R-MEWS or eCART or CEWS or TREWScore or DENWIS or DTEWS or DMEWS or POTTS or PAR-T or ViEWS-L).ti,ab.

38. (develop$ or design$ or creat$ or build$ or contruct$ or validat$).ti,ab.

39. Validation Studies.pt.

40. 38 OR 39

41. 36 AND 40

42. 37 AND 40

43. 41 OR 42

44. 24 OR 43

45. ((child OR infant OR pediatrics) NOT adult).sh.

46. 44 NOT 45
